# A novel inhibitory BAK antibody enables assessment of non-activated BAK in cancer cells

**DOI:** 10.1038/s41418-024-01289-3

**Published:** 2024-04-06

**Authors:** Hema Preethi Subas Satish, Sweta Iyer, Melissa X. Shi, Agnes W. Wong, Karla C. Fischer, Ahmad Z. Wardak, Daisy Lio, Jason M. Brouwer, Rachel T. Uren, Peter E. Czabotar, Michelle S. Miller, Ruth M. Kluck

**Affiliations:** 1 Walter and Eliza Hall Institute of Medical Research, 1G Royal Parade, Parkville, VIC 3052 Australia; 2 Department of Medical Biology, The University of Melbourne, Parkville, VIC 3010 Australia; 3 Present Address: Vanderbilt Vaccine Center, Vanderbilt University Medical Center, Nashville, TN 37232 USA

**Keywords:** Protein folding, X-ray crystallography, Cancer

## Abstract

BAX and BAK are pro-apoptotic members of the BCL2 family that are required to permeabilize the mitochondrial outer membrane. The proteins can adopt a non-activated monomeric conformation, or an activated conformation in which the exposed BH3 domain facilitates binding either to a prosurvival protein or to another activated BAK or BAX protein to promote pore formation. Certain cancer cells are proposed to have high levels of activated BAK sequestered by MCL1 or BCLX_L_, thus priming these cells to undergo apoptosis in response to BH3 mimetic compounds that target MCL1 or BCLX_L_. Here we report the first antibody, 14G6, that is specific for the non-activated BAK conformer. A crystal structure of 14G6 Fab bound to BAK revealed a binding site encompassing both the α1 helix and α5-α6 hinge regions of BAK, two sites involved in the unfolding of BAK during its activation. In mitochondrial experiments, 14G6 inhibited BAK unfolding triggered by three diverse BAK activators, supporting crucial roles for both α1 dissociation and separation of the core (α2-α5) and latch (α6-α9) regions in BAK activation. 14G6 bound the majority of BAK in several leukaemia cell lines, and binding decreased following treatment with BH3 mimetics, indicating only minor levels of constitutively activated BAK in those cells. In summary, 14G6 provides a new means of assessing BAK status in response to anti-cancer treatments.

## Introduction

**Fig. 1:**
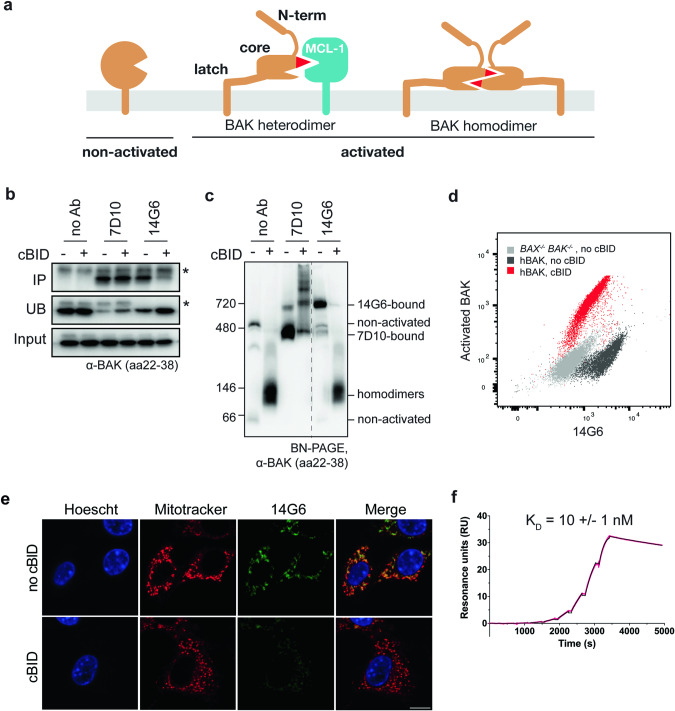
The 14G6 antibody binds specifically to non-activated BAK. **a** Diagram of three BAK conformations present in cells. Non-activated BAK is anchored in the mitochondrial outer membrane via a C-terminal transmembrane domain (α9) [53], with the remaining 8 helices forming a globular protein with hydrophobic surface groove [44]. Upon activation, the N-terminus (α1) and latch (α6-α8) separate from the core (α2–α5). Activated BAK can be sequestered by prosurvival proteins (e.g. MCL1) or can pair up as homodimers capable of pore formation. **b** 14G6 immunoprecipitates BAK only prior to its activation by cBID. Membrane fractions from *bak*^*−/−*^*bax*^*−/−*^ MEFs expressing hBAK were incubated with or without cBID (30 °C, 30 min). Samples were then solubilized in 1% digitonin and immunoprecipitated with no antibody or with 7D10 or 14G6. The immunoprecipitated (IP), unbound (UB) and input fractions were immunoblotted for BAK (clone aa23-38). Data are representative of two independent experiments. **c** 14G6 gel-shifts BAK only prior to its activation by cBID. Membrane fractions treated as in (**b**) were moved to ice and incubated with the indicated antibodies for 30 min, solubilized with 1% digitonin and run on blue-native PAGE (BN-PAGE) before immunoblotting for BAK (clone aa23–38). On BN-PAGE, non-activated BAK (lane 1) tends to run as a monomer (~66 kD) or in a complex with VDAC2 (~480 kD) [55]. Note that 7D10 gel-shifts BAK both before and after its activation, and when bound to BAK homodimers can generate a ladder indicating homodimers are in clusters. Note also that 14G6-bound BAK routinely runs at a significantly higher molecular weight (~720 kD) than 7D10 bound to non-activated BAK (~480 kD). Dashed line indicates deletion of lanes from the gel. Data are representative of two independent experiments. **d** 14G6 specificity for non-activated BAK on flow cytometry. Membrane fractions treated as in (**b**) were stained with 14G6 and with the G317-2 antibody to activated BAK prior to analysis by flow cytometry. (Also see Fig. S1a). Data are representative of two independent experiments. **e** 14G6 specificity for non-activated BAK on immunocytochemistry. Membrane fractions treated as in (**b**) were stained with 14G6-AF488, Mitotracker Deep Red and Hoescht prior to analysis by immunocytochemistry. Bar, 5 μm. Data are representative of two independent experiments. **f** 14G6 binds to BAK with a *K*_D_ of 10 nM as determined by Surface Plasmon Resonance. Kinetic parameters were determined by capturing 14G6 on a Protein A chip and injecting a threefold dilution series of BAK in a single-cycle kinetic method (0, 0.45, 1.4, 4, 12, 37, 111, 333, 1000 nM). *K*_D_ is the average of three independent experiments ± standard deviation. Sensorgram shown is representative of these experiments. Raw data are shown in pink, fitted curve in black.

The mitochondrial pathway of apoptosis is regulated by the BCL2 family of proteins that includes prosurvival proteins (e.g. BCL2 and MCL1) and two sets of pro-apoptotic proteins, the BH3-only proteins (e.g. BIM, BID) and the pore-forming proteins (BAX, BAK and BOK) [1, 2]. Apoptosis is initiated by upregulation of the BH3-only proteins which can bind to prosurvival members, but can also trigger BAX and BAK to unfold and convert into an activated conformation. Activated BAX and BAK can be sequestered by prosurvival proteins or can pair up as homodimers capable of pore formation (Fig. 1a). Thus, for BCL2 signalling to achieve mitochondrial pore formation and subsequent cell death depends on protein levels and protein-protein interactions, as well as on the BAX and BAK activation status.

From a structural point of view, several features of BAX and BAK unfolding into their activated conformations to form heterodimers and homodimers have been identified. Activator BH3-only proteins (e.g. BIM) can bind to a hydrophobic surface groove (α3–α5) on the BAX and BAK proteins [3–6] to trigger major BAX and BAK unfolding. This activation process includes dissociation of α1 [7–9], and separation of α2–α5 core from the α6-α9 latch [4, 5]. The resulting exposure of the BH3 domain (in α2) allows reciprocal BH3:groove interactions with a second activated BAX or BAK molecule to generate stable symmetric homodimers (Fig. 1a) [10–12]. Homodimers cluster on the mitochondrial outer membrane to trigger membrane permeabilization and release apoptogenic factors such as cytochrome *c* [13–16]. Binding of BAX and BAK by prosurvival proteins is less well characterized, but involves capture of the exposed BH3 domain of activated BAX or BAK in the hydrophobic groove of a prosurvival protein (Fig. 1a) [17, 18].

During apoptosis in cells, BAX and BAK activation status can be assessed via a range of techniques including epitope exposure, limited proteolysis, co-immunoprecipitation, Blue-Native PAGE, site-directed spin labelling and size exclusion chromatography [19–23]. Antibodies to N-terminal epitopes recognize all forms of activated BAX or BAK (i.e. homodimers and heterodimers both display a solvent-exposed N-terminus) [7–9]. What has been lacking to date are antibodies that can distinguish between heterodimers and homodimers, or that specifically bind the non-activated BAK conformer.

Consideration of BAX and BAK activation in the context of BCL2 signalling in cells has resulted in several models. Here, those models will be referred to as “BAX/BAK cell death models” to differentiate them from BAX and BAK activation defined by structural unfolding. These models fall into two broad classes: the direct and the indirect cell death models. In the “direct” BAX/BAK cell death model, BAX and BAK are in the non-activated conformation in healthy cells, and become activated by BH3-only proteins [24]. In the “indirect” model, BAK (and BAX) are constitutively activated and sequestered by prosurvival proteins, and then competed off by upregulated BH3-only proteins [25]. Elements of both direct and indirect models may occur according to the unified, embedded and interconnected cell death models [26–29]. In the recent “membrane-mediated spontaneous” model, BAX and BAK can convert to the activated conformations upon translocation to the mitochondrial outer membrane [30].

BAX and BAK status is of particular interest to cancer treatment as most anti-cancer therapies aim to achieve mitochondrial permeabilization (by BAX or BAK) that efficiently induces apoptosis. For example, BAX mutations can cause resistance to BH3 mimetic based therapies in leukaemia patients [31, 32]. Assessment of BAK activation status in leukaemia and ovarian cancer cell lines found that up to 80% of BAK was constitutively activated and bound to prosurvival proteins as heterodimers [33, 34], an example of the indirect BAX/BAK cell death model. Moreover, those heterodimers predicted responses to BH3 mimetics and other anti-cancer agents. Finally, insufficient BAX/BAK function can result in sublethal cytochrome *c* release and DNA damage as well as cells with a persister phenotype [35–37]. Thus, improved means of measuring BAX and BAK status, before and after anti-cancer treatments, may help improve patient responses.

In addition to BAX and BAK regulation by BCL2 signalling, antibodies that bind directly to BAX and BAK can regulate their function. Antibodies that activate BAK (or the mitochondria-targeted BAX variant S184L) bind to the N-terminal α1-α2 loop region to trigger activation-associated conformational rearrangements [38–40]. Antibodies that inhibit BAX prevent its translocation [39] or obstruct the N-terminal activation site [41].

Here we identify and characterize the first antibody (clone 14G6) specific for the non-activated conformation of BAK. The antibody binds with nanomolar affinity and prevents BAK activation triggered by three distinct activators: cBID, a BAK-activating antibody, and heat. A crystal structure revealed that 14G6 embraces a complex non-linear epitope of both the α5–α6 hinge region and the α1 helix in non-activated BAK, supporting previous evidence that activation requires separation of the latch and the α1 helix from the α2-α5 core. In several untreated leukaemia cell lines 14G6 antibody recognized the majority of BAK, indicating minimal constitutively activated BAK before apoptotic signalling. Thus, this antibody provides a new tool for assessing BAK activation status both before and after anti-cancer treatments.

## Results

### The 14G6 antibody specifically binds to non-activated BAK

To understand the major conformational changes involved in BAX and BAK activation and oligomerization, we and others have generated several antibodies to different regions in the two proteins [10, 38, 40]. To generate additional antibodies for interrogating BAK-mediated apoptosis, mice were immunized with human BAK lacking endogenous cysteine residues, the flexible N terminus and the C terminal transmembrane anchor (BAKΔN22ΔC25Δcys, herein referred to as BAKΔTM). Using mouse B cell cloning, B cells that bind to BAKΔTM were selected and the recovered antibody variable regions expressed as chimeras containing human constant regions. When tested by ELISA, several antibodies were found to bind BAKΔTM.

When tested for binding to full-length BAK in mitochondria, the 14G6 clone showed specificity for non-activated BAK (Fig. 1b–e). These experiments were performed using *bak*^*−/−*^*bax*^*−/−*^ mouse embryonic fibroblasts (MEFs) stably expressing human BAK, and the cell membrane permeabilized with digitonin to allow access of cBID and antibodies. Immunoprecipitation showed that clone 14G6 could bind BAK before but not after incubation with cBID (Fig. 1b). In contrast, clone 7D10, that binds all forms of BAK [38, 40] could bind BAK both before and after BAK activation by cBID (Fig. 1b). The same pattern of antibody binding was shown by blue-native PAGE (BN-PAGE), with 7D10 binding indicated by its ability to shift BAK to a higher molecular weight native complex (gel-shift) both before and after cBID treatment, while 14G6 gel-shifted BAK only prior to cBID (Fig. 1c). When tested on flow cytometry and immunocytochemistry, 14G6 also showed high staining before but not after BAK activation by cBID (Figs. 1d, e and S1a). Surface Plasmon Resonance (SPR) analysis of 14G6 with BAKΔTM showed tight binding (*K*_D_ of 10 ± 1 nM) with a slow dissociation rate (Fig. 1d; Table 1). These data, together with the failure of 14G6 to bind to any BAK peptide on peptide array (Fig. S1b), indicate that 14G6 binds to a non-linear epitope, and is specific for the non-activated conformer of BAK.

**Table 1:** 14G6 binding characteristics by SPR.

	*k*_a_ (1 × 10^3^/Ms)	*k*_d_ (1 × 10^−5^/s)	*K*_D_ (nM)
**hBAK**	6 ± 2	7 ± 2	10 ± 1
**hBAK M60G**	110 ± 10	7 ± 1	0.6 ± 0.2
**hBAK M60W**	4.9 ± 0.7	5 ± 1	9 ± 3
**mBAK**	0.81 ± 0.05	92 ± 2	1140 ± 90

### 14G6 prevents BAK activation

**Fig. 2:**
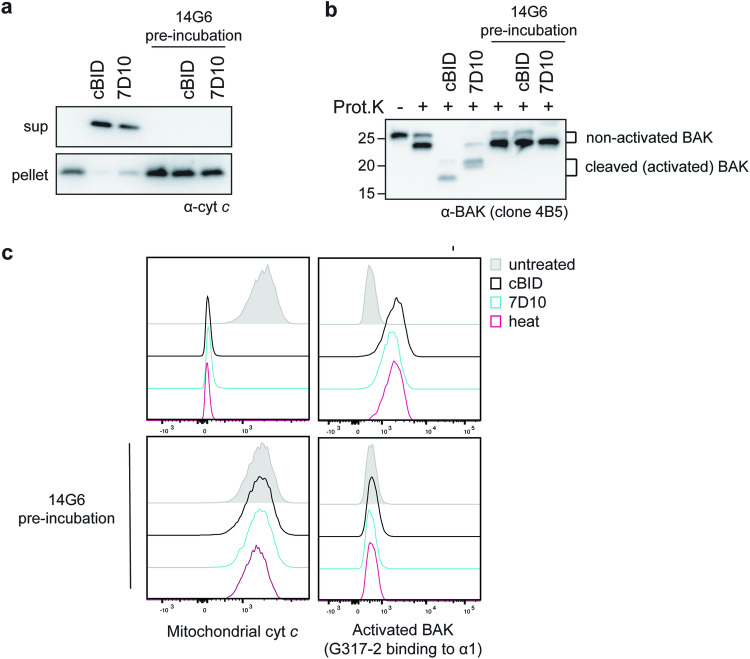
14G6 prevents BAK activation and cytochrome *c* release. **a** 14G6 blocks cytochrome *c* release triggered by cBID or 7D10. *bak*^*−/−*^*bax*^*−/−*^ MEFs expressing hBAK were permeabilized and incubated with or without 14G6 (on ice, 10 min) prior to incubation with or without cBID or 7D10 (30 °C, 30 min). Samples were then centrifuged and supernatant and pellet fractions immunoblotted for cytochrome *c*. Data are representative of two independent experiments. **b** 14G6 inhibits unfolding of BAK as shown by limited proteolysis. The pelleted mitochondrial fractions from (**a**) were treated with proteinase K and immunoblotted with antibody to the BAK BH3 domain (clone 4B5). After proteinase K, non-activated BAK runs as an ~23 kD fragment (lane 2), while cBID-activated BAK is seen as a ~16 kD fragment (lane 3) due to cleavage in the α1–α2 loop, and 7D10-activated BAK runs as a slightly larger fragment as 7D10 masks one of the cleavage sites [19]. Data is representative of two independent experiments. **c** 14G6 blocks BAK activation and cytochrome *c* release triggered by three distinct stimuli. *bak*^*−/−*^*bax*^*−/−*^ MEFs expressing hBAK were permeabilized and incubated with or without 14G6 prior to incubation with or without cBID or 7D10 as indicated. Additional aliquots were also incubated at 43 °C (heat) to activate BAK. Cells were fixed and stained for cytochrome *c* or for activated BAK (clone G317-2) and analyzed by flow cytometry. Data are representative of at least two independent experiments.

We next asked if 14G6 might activate BAK as does the 7D10 antibody [38], or if it might prevent BAK activation. Permeabilized cells were incubated with 14G6, with or without subsequent incubation with cBID or 7D10 (Fig. 2a, b). 14G6 was able to block cytochrome *c* release initiated by cBID or 7D10 (Fig. 2a) and to block BAK conformation change assessed by the ability of proteinase K to cleave in the α1–α2 loop (Fig. 2b). When assessed by flow cytometry, 14G6 was again found to block cytochrome *c* release and BAK activation after treatment with cBID or 7D10 as well as heat (Fig. 2c). In these experiments, 7D10 could still bind BAK that was bound by 14G6, as shown by gel-shift on BN-PAGE (Fig. S2).

### Structural characterization of 14G6 Fab binding to BAK reveals non-linear epitope

**Fig. 3:**
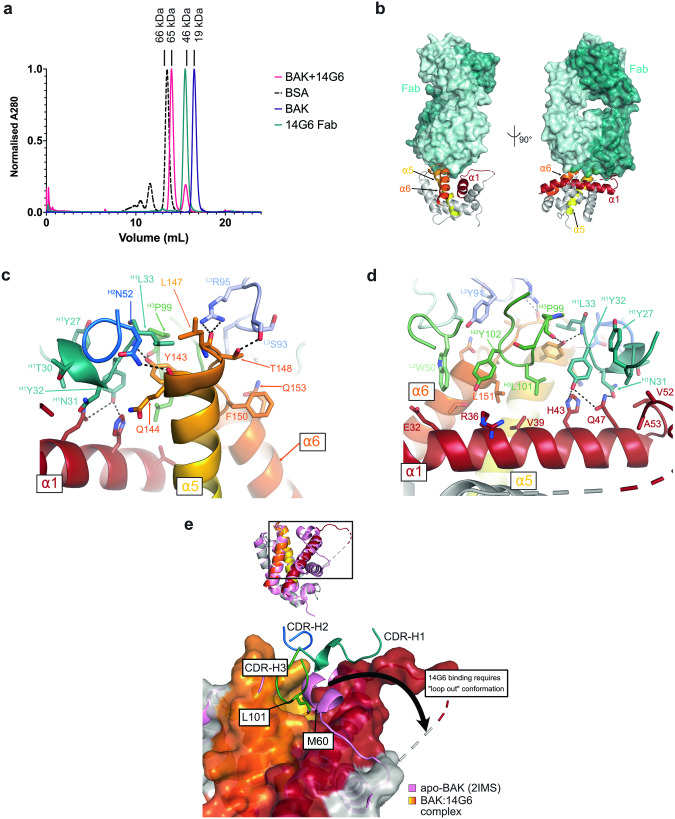
Crystal structure of 14G6-bound human BAK reveals mechanism of inhibition. **a** Size exclusion chromatography demonstrates that 14G6 Fab forms a 1:1 complex with BAK. BSA, bovine serum albumin, MW 66 kDa. **b** 14G6 binds BAK via interactions with the α5-α6 hinge (α5 and α6 shown in yellow and orange, respectively) and α1 (shown in red). The light and heavy chains of the Fab are shown in light and dark teal, respectively. **c**. Detailed interactions between 14G6 and BAK α5-α6 hinge. The light chain complementarity determining regions (CDRs) are shown in pale cyan (L1), light green (L2) and light blue (L3), and the heavy chain CDRs are shown in teal (H1), blue (H2), green (H3). **d** Detailed interactions between 14G6 and BAK α1. Colours as per (**c**). **e** 14G6 binding displaces the α1-α2 loop and inserts CDR-H3 L101 into the M60 pocket. apo-BAK (2IMS, pink) with closed α1-α2 loop overlaid with 14G6-bound BAK (grey, yellow, orange, red, chain D) showing open, unresolved loop.

To identify the 14G6 epitope we determined the crystal structure of the 14G6 Fab bound to BAKΔTM. The 14G6 Fab was generated by papain cleavage of the full-length antibody and purified by cation exchange. The 14G6 Fab: BAKΔTM complex runs as a monodisperse complex on size exclusion chromatography, with a molecular weight of *ca* 65 kDa (Fig. 3a).

The asymmetric unit of the crystal contains two copies of the 14G6 Fab: BAK complex (Table 2). The shape complementarity metric Sc is 0.617 (over the trimmed surface area of 534 Å^2^), which is typical of an antibody-antigen interface [42, 43]. 14G6 binds BAK by cradling the α5-α6 hinge region and making contacts with BAK α1 (Fig. 3b). Binding at the α5-α6 hinge is mediated by all three heavy chain CDR loops and CDR-L2 and L3 (Fig. 3c). The backbone of CDR-H1 L33 and CDR-H3 P99 interacts with BAK Y143, while the side-chain carboxamide of CDR-H2 N52 interacts with the backbone carbonyl of BAK Q144. The side chains of CDR-L3 S93 and R95 interact with BAK T148 hydroxyl and L147 backbone carbonyl, respectively, while CDR-L2 W50 makes a hydrophobic contact with the end of α6 (Fig. 3d).

**Table 2:** Data collection and refinement statistics for BAK complex with the Fab fragment of antibody 14G6 (PDB entry 8UKY).

**Data collection**	
Space Group	P1
Cell dimensions	
* a*, *b*, *c* (Å)	71.55, 72.62, 75.28
* α*, *β*, *γ* (°)	103.0, 103.9, 112.2
Resolution (Å)	45.38–2.40 (2.48–2.40)
Total reflections	106,456 (10837)
Unique reflections	48,287 (4806)
* R*_merge_	0.066 (0.44)
* R*_pim_	0.056 (0.37)
* I/σ*(*I*)	9.6 (1.8)
* CC*_1/2_	0.997 (0.819)
Completeness (%)	96.4 (95.4)
Redundancy	2.2 (2.3)
**Refinement**	
No. reflections	48,209 (4802)
* R*_work_/*R*_free_	0.215/0.252 (0.322/0.347)
No. atoms	9337
Protein	8914
Ligand	150
Water	273
* B* factors (Å^2^)	
Protein	54.9
Ligand	69.3
Water	43.8
R.m.s. deviations	
Bond lengths (Å)	0.004
Bond angles (°)	0.94

The BAK α1-α2 loop has been displaced from its usual position and the residues are mostly unresolved in the electron density, suggesting some flexibility (Fig. 3e). This loop movement allows the antibody to interact directly with α1 through its two heavy chain CDR loops (Fig. 3d). CDR-H1 N31 and Y32 both form hydrogen bonds with BAK Q47, with additional hydrophobic contacts extending along the C-terminal half of α1. CDR-H3 L101 has inserted itself into the pocket usually occupied by the linchpin residue M60 [40], making hydrophobic contacts with V39 and H43 (Fig. 3d). Thus CDR-H1 and CDR-H3 may act to stabilize BAK α1 in the closed conformation.

BAK is in the non-activated conformation as shown in previous structures, with an RMSD of 0.72 Å (chain C) −0.76 Å (chain D) when aligned with 2IMS [44]. There are a few noticeable differences between the structures of 2IMS and 14G6-bound BAK (Fig. S3a, b). In the 14G6-bound structure, α3 is extended at both the N- and C-terminal ends, with the α3-α4 loop displaying a different conformation compared with 2IMS. This results in a more closed BH3 binding groove (Fig. S3a, b). However, the altered conformation of α3 may be influenced by crystal contacts, since BAK H99 (at the α3 C-terminus) in chain D makes a hydrogen bond with the antibody light chain from the other copy of the complex (Fig. S3c).

### Mechanism of inhibition – mutations in BAK N-terminus suggest a role for binding to the α1 region

The structure of 14G6-bound to BAK suggests three possible mechanisms of inhibition. Firstly, the closed groove may prevent BH3-only proteins from binding (Fig. S3a, b). Second, contact with α1 may prevent dissociation of this helix early on in BAK activation. Lastly, contact with the α5–α6 hinge may prevent unlatching of the α2–α5 core from α6–α8. To test whether 14G6-bound BAK could still bind BH3-only proteins, we used a mutant of BAKΔTM with a cysteine in the groove (K113C), along with a cysteine-mutant of BID (R84C) and observed the BAK band shift on non-reducing SDS-PAGE. The covalent linkage between BAK and BID forms even in the absence of oxidizing agent (Fig. S3d). This link can still form even when BAK is pre-incubated with excess 14G6, suggesting that 14G6 binding does not prevent BID from binding to BAK and that the groove can adopt both open and closed conformations when bound to 14G6. Thus, 14G6 binding appears not to limit binding of either cBID (Fig. S3d) or the activating antibody 7D10 (see Fig. S2).

We next tested mutation of the BAK M60 residue, to assess if 14G6 binding requires displacement of the α1–α2 loop (Fig. 3e). Our recent study showed that mutagenesis of the α1–α2 loop (M60G) could not only “open” the α1-α2 loop, but could also promote dissociation of α1 (and BAK activation) [40]. Accordingly, SPR analysis of BAK M60G binding to 14G6 resulted in a stronger binding affinity (0.6 nM), primarily due to an increase in the association rate by 18-fold (Table 1, Fig. S4d). We had also found that mutation of M60 to a tryptophan appeared to “close” the loop and increased the thermal stability of BAK and reduced spontaneous activation [40]. Surprisingly, binding of 14G6 to BAK M60W showed no significant difference in kinetics or affinity compared with the WT (Table 2, Fig. S4e). Although lower spontaneous activity in the M60W mutant does suggest that the loop exists more often in the closed orientation or that the threshold for releasing the loop is increased, perhaps neither of these are significant enough to alter the binding kinetics of 14G6. Another potential explanation is that 14G6 binds to the α5–α6 hinge and then induces the movement of the α1–α2 loop, and that the threshold for this induced movement is similar in the M60W mutant compared with wild-type.

### 14G6 binds mouse BAK with lower affinity

**Fig. 4:**
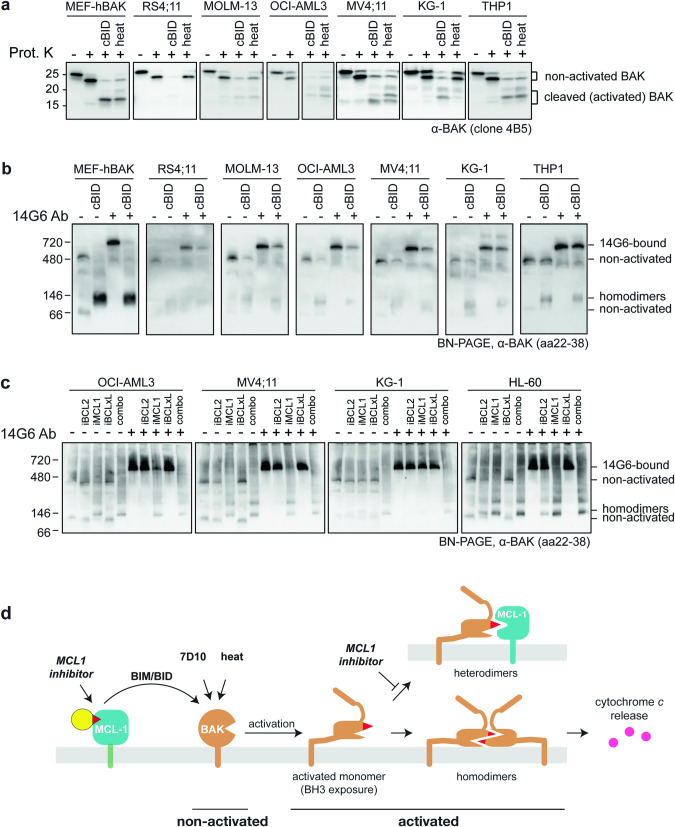
BAK is not constitutively activated in blood cancer cell lines. **a** The majority of BAK in untreated leukaemia cell lines is resistant to proteinase K. *bak*^*−/−*^*bax*^*−/−*^ MEFs expressing hBAK and the indicated cancer cell lines were permeabilized and incubated with cBID (cBIDM97A) or incubated at 43 °C (heat) to activate BAK. Membrane fractions were incubated with proteinase K and blotted for BAK (as in Fig. 2b). Data are representative of at least two independent experiments. **b** The majority of BAK in untreated leukaemia cell lines is gel-shifted by 14G6. Membrane fractions from (**a**) were incubated with or without 14G6, run on BN-PAGE, and blotted for BAK (as in Fig. 1c). Data are representative of at least two independent experiments. **c** BAK activation by BH3 mimetic treatment demonstrated by loss of 14G6 binding. Four acute myeloid leukaemia cell lines were incubated with 1 µM iBCL2 (venetoclax), iMCL1 (S63845), iBCLxL (A-1331852), alone or in combination for 3 h. Membrane fractions were then incubated with or without 14G6, and analyzed as in (**b**). Cell lysates were also assessed for BAK and BAX cleavage by proteinase K (Fig. S6). Data are representative of at least two independent experiments. **d** Schematic of BAK activation by various stimuli demonstrating that in the cell types examined, the majority of BAK becomes activated only after apoptotic signalling. Here, MCL1 represents prosurvival proteins, as MCL1 inhibitor alone could trigger BAK activation in three cell lines (Figs. 4c and S6).

Mouse and human BAK share >90% sequence identity and have a high degree of structural similarity (RMSD 0.617 Å between 2IMS and 6MCY) (Fig. S5a, b). As such, while 14G6 was raised against human BAK, we wondered whether it might also bind mouse BAK. We found that 14G6 readily bound and inhibited human BAK when pre-incubated with membrane fractions on ice (Figs. 1–4). In contrast, pre-incubation at 30 °C was required for efficient 14G6 binding (Fig. S5c) and inhibition of mouse BAK (Fig. S5d). To rationalize this difference, we determined the kinetic and affinity parameters by SPR and found that 14G6-bound mouse BAK with a *K*_D_ of 1.14 µM, which is 100-fold lower than human BAK (Table 1, Fig. S4f). This is due to changes in both the association (0.81 × 10^3^ 1/Ms, tenfold slower) and dissociation (9.2 × 10^−4^ 1/s, tenfold faster) rates.

Despite the 100-fold difference in affinity, the epitope is essentially conserved within both human and mouse BAK. The single amino acid difference in the region that interacts with 14G6 is hBAK E50 which makes a charge-assisted hydrogen bond to CDRH1-N31 on 14G6, whereas in mBAK this residue is a glutamine. This subtle change may contribute to the faster dissociation rate observed for 14G6-mBAK interactions. Outside of the direct interaction region, the mBAK sequence differences may contribute to the lower affinity. For example, in mBAK there is a hydrogen bond between mD59 (α1–α2 loop) and mQ45 (α1) that is not present in hBAK and this may stabilize the α1–α2 loop in the closed conformation compared to hBAK, which cannot form this hydrogen bond (V61 and Q47, respectively) (Fig. S5b). This may account for the slower on-rate observed for 14G6-mBAK interactions. There are also differences in amino acids in α3, α4, α5 and α6 (Fig. S5a, b) that despite not directly contacting 14G6, may have (difficult to predict) allosteric effects or effects on flexibility that promote the lower binding affinity observed for mBAK.

### 14G6 as a new tool to assess BAK status in cancer cells

Due to the specificity of 14G6 for non-activated BAK, this antibody provides a new means of assessing BAK status in each cell context. In MEFs, the majority of BAK is not constitutively activated based on 14G6 binding by immunoprecipitation and gel-shift (Fig. 1b, c), and on the lack of cleavage by proteinase K (lane 2, Fig. 2b). Although less quantitative, single cell analysis also indicated the presence of significant non-activated BAK in untreated MEFs, as 14G6 binding decreased after cBID treatment (Fig. 1d, e, S1a). In contrast, up to 80% of BAK was found to be constitutively activated (i.e. forming heterodimers with prosurvival proteins) in certain cell lines, including MV4;11 and HL-60 acute myeloid leukaemia cells [33, 34].

To examine if constitutively activated BAK is common in cancer cell lines, a series of blood cancer cell lines established from patients with acute lymphoblastic leukaemia (ALL) or acute myeloid leukaemia (AML) were examined for BAK activation status using limited proteolysis (Fig. 4a) or 14G6 gel-shift (Fig. 4b). If a significant portion of BAK was constitutively activated, BN-PAGE would show minimal gel-shift by 14G6 both before and after apoptotic signalling. However, in each case, only minimal BAK was constitutively activated as most BAK was cleaved only to the ~23 kD band (lane 2; Fig. 4a), and could be gel-shifted by 14G6 (lane 3 vs lane 1; Fig. 4b). After treatment with cBID or heat, BAK became activated as shown by cleavage to smaller fragments (lanes 3 and 4; Fig. 4a) and less gel-shift by 14G6 (lane 4 vs lane 3; Fig. 4b). Thus, minimal constitutively activated BAK is present in these cells.

To test BAK activation in response to BH3 mimetics, four cell lines were incubated with inhibitors specific for BCL2, MCL1 or BCLxL, alone or in combination. A combination of inhibitors was able to activate the majority of BAK in each cell line, as 14G6 was no longer able to gel-shift BAK (lane 10 vs lane 6; Fig. 4c). Moreover, BAK (and BAX) became susceptible to limited proteolysis (lane 6 vs lane 2; Fig. S6). The MCL1 inhibitor alone could trigger BAK and BAX activation in three cell lines (Figs. 4c and S6). Thus, in these cells, BH3 mimetics are not acting by displacing constitutively activated BAK from prosurvival proteins. Rather, BH3 mimetics displace BH3-only proteins which then activate BAK (Fig. 4d).

## Discussion

Since BAX and BAK were shown to be essential for apoptotic cell death [45], these proteins have been widely studied [1]. In their non-activated forms, BAK and BAX form a globular bundle of 8 or 9 helices. Upon activation, the proteins partially unfold and form complexes with like molecules to form homodimers, or with prosurvival proteins to form heterodimers (Fig. 1a). Each cell may contain a mixture of the three conformers dependent on the balance of BCL2 family members present in each cell and the cell stresses encountered. Determining the proportion of each conformer in individual cells is not yet possible, but is required to better understand responses to anti-cancer therapies such as the BH3 mimetics.

We report the generation and characterization of 14G6, the first antibody specific for the non-activated form of BAK. Specificity is explained by 14G6 being a conformational antibody with a binding site encompassing two regions, α1 and the α5–α6 hinge, both of which undergo major changes upon BAK activation. Previous studies have shown that disulfide tethering of α5-α6 was able to block the unlatching leading to MOMP [5], with evidence that cBID could still bind and trigger partial α1 dissociation [7]. The requirement for α1 dissociation for BAK activation is consistent with a role for the BH4 domain in stabilizing the non-activated protein [7, 46]. By binding to both the α5-α6 hinge and α1, 14G6 appears able to prevent both unlatching and α1 dissociation, maintaining BAK in a fully non-activated state. Accordingly, 14G6 binding inhibited BAK activation, as addition of 14G6 to mitochondria-enriched membranes inhibited BAK-mediated cytochrome *c* release initiated by three distinct BAK activators, the BH3-only protein cBID, the 7D10 antibody, and mitochondrial incubation at 43 °C. While many inhibitory antibodies act by preventing activators from binding to their activation sites, 14G6 did not prevent binding of either cBID or 7D10, but bound to BAK at two sites critical for its unfolding.

No other known antibodies to BAK involve an epitope spanning two distinct binding sites [7]. There is, however, such an antibody (3G11) to BAX whose epitope is similar to that of 14G6 [41]. Although high-resolution structural information is not available for the BAX-3G11 complex, hydrogen-deuterium exchange mass-spectrometry (HDX-MS) identified the epitope as involving residues along the length of α1 and α6, along with some residues at the C-terminal end of α5, suggesting binding at the hinge region of α5–α6, similar to 14G6. A key difference, however, is that 3G11 appears to bind directly to the BAX α1–α2 loop, possibly locking it in place [41], while 14G6 displaces the α1–α2 loop and stabilizes BAK in its loop-open inactive conformation.

Displacement of the BAK α1–α2 loop allowed direct binding of 14G6 to α1. Similar displacement was evident in the structure of BAK bound by the 7D10 activating antibody, whose epitope was in the α1-α2 loop itself [40]. 7D10 binding removes M60 (within the α1–α2 loop) from its pocket (involving α1) to promote α1 dissociation and BAK activation [40]. In the 14G6 crystal structure, 14G6 appears to substitute for M60 by inserting Leu101 from CDR-H3 into the M60 pocket to bind directly to α1 and hold it in place to prevent BAK activation. Thus, structures of the 14G6 inhibiting antibody and the 7D10 activating antibody together reveal molecular events involved in BAK N-terminus exposure during apoptosis.

Inhibition by 14G6 shares a common theme to two other reported BAK inhibitors, despite binding at distinct sites. The BH3 peptide inhibitor of BAK, Bim-H3Pc-RT, binds at the canonical groove binding site, where a pentyl-carboxylate side chain inserts into a pocket to make a key charge-charge interaction with R42 on α1, thus stabilizing the helix and preventing α1 dissociation during activation [47]. This interaction also contributes to specificity for human BAK, as the corresponding residue in mouse BAK is L40. A small-molecule inhibitor of BAK activation that is specific for mouse BAK has also been described [48]. It binds the BAK-VDAC2 complex, enhancing VDAC2’s ability to restrain BAK in a non-activated state. Chimera experiments identified residues in α1 and α9 as being important for activity, specifically mouse BAK L40 (human R42) [48–51]. This small-molecule inhibitor prevents α1 exposure. While each of the inhibitors binds at a distinct site, either directly or indirectly influencing BAK, they all function by blocking major structural rearrangements, namely, α1 dissociation and α5-α6 hinge unlatching.

As the first antibody specific for non-activated BAK, 14G6 provides a new means of characterizing BCL2 family signalling in cells, complementing current BAK activation assays [19–23]. 14G6 specificity is evident in both bulk and single cell assays, and in the presence or absence of 1% digitonin. The antibody binds mouse as well as human BAK, although with lower affinity. The use of 14G6 in single cell analyses can address questions that bulk assays cannot. For example, in flow cytometry 14G6 can be multiplexed with other antibodies (Figs. 1d and S1a) with the potential to identify heterogeneity in patient samples and their response to therapy. In single cell imaging, 14G6 may identify heterogeneity of BAK activation within a cell.

Here we used 14G6 binding in bulk assays to quantitate the proportion of BAK in the non-activated conformation prior to apoptotic signalling. 14G6 gel-shift showed that the majority of BAK is in the non-activated conformation in six cancer cell lines as well as in SV40-immortalized MEFs (Fig. 4). This contrasts with reports of up to 80% constitutively activated BAK in certain leukaemia and ovarian cancer cell lines [33, 34]. In those studies, spurious BAK activation by detergents appeared not to be involved, as size exclusion identified constitutively activated BAK oligomers after extraction with either CHAPS or digitonin [33], detergents generally considered unable to unfold BAK. Our findings also provide no evidence for membrane-mediated activation of BAK [30, 52] as non-activated BAK is present in the mitochondria-enriched membrane fraction in all cells examined (Fig. 4a,b and S6), and in MEFs was shown to be membrane-integrated via a C-terminal transmembrane domain [53].

In the cells examined here, BAK binding to prosurvival proteins must occur after apoptotic signalling (including BH3 mimetic treatments), and contribute to resistance (Fig. 4d) rather than to sensitization [33, 34]. Hence, BH3 mimetics principally act upstream to compete off BAK-activating BH3-only proteins, as well as prevent sequestration of activated BAK (Fig. 4d). Whether BH3 mimetics can also compete off BAK from MCL1 or BCLX_L_ is less clear, with evidence that such heterodimers are relatively insensitive to BH3 mimetics [29, 54]. These findings suggest that measuring interactions between BCL2 family members in individual cells may help clarify mechanisms of resistance to BH3 mimetics and other cancer treatments.

## Methods and materials

### Cell lines

Mouse embryonic fibroblasts (MEFs) derived from *bax*^*−/−*^ or *bak*^*−/*−^*bax*^−*/−*^ C57BL/6 mice were transformed with SV40 as previously described [10, 55]. For cells expressing human BAK, full-length BAK was stably expressed in SV40-immortalized *bak*^*−/−*^*bax*^*−/−*^ mouse embryonic fibroblasts (MEFs) [38]. MEFs were maintained in Dulbecco’s modified Eagle’s medium (DMEM) supplemented with 10% foetal bovine serum, 0.1 mM L-asparagine and 55 μM 2-mercaptoethanol at 37 °C in a humidified 10% CO_2_ incubator, and regularly tested negative for mycoplasma (Lonza MycoAlert).

The human ALL cell line RS4;11 [56] was originally imported to WEHI from ATCC (American Type Culture Collection;VI USA). Human AML cell lines MOLM-13 [57], KG-1 [58], HL-60 [59] and OCI-AML3 [60] were originally imported to WEHI from DSMZ (Deutsche Sammlung von Mikroorganismen unde Zellkulturen, Leibniz, Germany), and MV4;11 [61] and THP-1 [62] from ATCC (American Type Culture Collection;VI USA). RS4;11, MOLM-13, KG-1 and HL-60 cell lines were cultured in RPMI-1640 medium (Gibco #31800089) supplemented with 10% heat-inactivated FCS, 100 U/mL penicillin and 100 µg/mL streptomycin (Gibco #15140122). MV4;11, and THP-1 cell lines were cultured in RPMI-1640 medium supplemented with 15% heat-inactivated FCS, 100 U/mL penicillin and 100 µg/mL streptomycin. The OCI-AML3 cell line was cultured in MEM alpha medium with GlutaMAX™ supplement (Gibco #32561037) supplemented with 10% heat-inactivated FCS (Sigma #F9423). All human leukaemia cell lines were maintained at 37 °C with 5% CO_2_ for <2 months, verified by STR profiling at the Australian Genomics Research Facility (AGRF) and regularly tested negative for mycoplasma.

### Preparation of mitochondria-enriched membrane fractions from MEFs and cancer cell lines

To generate mitochondria-enriched membrane fractions from MEFs and the human cancer cell lines, cells were spun and resuspended (at 1 × 10^7^ ml^−1^ for MEFs and THP-1 cells and at 3 × 10^7^ ml^−1^ for the smaller RS4;11, MOLM-13, OCI-AML3, MV4;11, KG-1 cells) in MELB buffer (93.5 mM sucrose, 20 mM HEPES, pH 7.4, 2.5 mM MgCl_2_ and 100 mM KCl) supplemented with Complete Protease Inhibitors (Roche, Castle Hill, NSW, Australia). Cell membranes were permeabilized by addition of 0.025% w/v digitonin and incubation on ice for 10 min, then the pellet (mitochondria-enriched membrane) fractions separated from the supernatant (cytosolic) fraction by centrifugation at 13,000 × *g* for 5 min. Membrane fractions were resuspended in MELB buffer supplemented with Complete Protease Inhibitors (Roche) and 8 μg/ml pepstatin A (Sigma; #26305-03-3).

### Mitochondria incubation and cytochrome *c* release assays

To trigger activation (unfolding) of BAK, mitochondria-enriched membrane fractions (50 μl) were incubated with 100 nM caspase-8-cleaved human BID (cBID) or human BIDM97A (cBIDM97A) or with 7D10 antibody (0.1 μg/μl) at 30 °C for 30 min. To test if 14G6 blocked BAK activation, membranes fractions were pre-incubated with 14G6 (0.1 μg/μl) on ice for 30 min. To monitor cytochrome *c* release from mitochondria, reactions were spun at 13,000 x *g* and the supernatant and pellet fractions immunoblotted for cytochrome *c*.

### BH3 mimetic treatment

For BAK activation by BH3 mimetics cells were supplemented with the broad spectrum caspase inhibitor Q-VD-OPh (MedChem Express, # HY-12305; 50 µM) for 30 min before addition of iBCL2 (Venetoclax/ABT-199, Active Biochem #A-1231), iMCL1 (S63845, Active Biochem #6044) and iBCLX_L_ (A-1331852, AbbVie, provided by G. Lessene) in dimethyl sulfoxide (DMSO), and incubated for a further 3 h. Either membrane fractions or whole cell lysates were assessed for BAK activation by proteinase K cleavage or 14G6 gel-shift on BN-PAGE as described.

### BAK activation assessed by limited proteolysis

BAK conformation change was assessed by limited proteolysis [19]. Briefly, 50 μl of membrane fractions resuspended in MELB with 8 μg/ml pepstatin A were pre-chilled to 0 °C then incubated with 30 μg/ml proteinase K (Sigma) on ice for 20 min. Reactions were stopped by addition of 1 mM phenylmethylsulfonyl fluoride, and samples immunoblotted with antibody to the BAK BH3 domain (clone 4B5) [19].

### Immunoprecipitation

Membrane fractions (at least 2.5 × 10^6^ cells per treatment) with or without cBID treatment were solubilized with 1% w/v digitonin in lysis buffer (20 mM Tris, 135 mM NaCl, 1.5 mM MgCl_2_, 1 mM EGTA, 10% glycerol, pH 7.4) on ice for 1 h. Lysates were centrifuged at 13,000 × *g* for 5 min and supernatants added to Protein G Sepharose and allowed to pre-clear at 4 °C for 1 h. To immunoprecipitate BAK, pre-cleared samples were added to fresh Protein G sepharose supplemented with 4 μg 7D10 rat monoclonal antibody or 14G6 mouse monoclonal antibody and incubated at 4 °C for 1–2 h. Unbound fractions were collected and the resin washed with lysis buffer containing 0.1% w/v digitonin, and bound protein eluted by boiling in sample buffer. Unbound and total lysates were immunoblotted for BAK.

### Blue-native PAGE of membrane fractions

BN-PAGE of extracted proteins was performed as previously described [63]. Cells or membrane fractions were solubilized in 20 mM Bis-Tris (pH 7.4), 50 mM NaCl, 10% glycerol, 1% (w/v) digitonin with 10 mM DTT before centrifugation at 13,000 × *g* to pellet insoluble debris.

### Western blotting

Immunoprecipitated samples and samples from mitochondrial assays were resolved by SDS-PAGE using Tris-Glycine gels (Bio-Rad) or by BN-PAGE using Bis-Tris gels (Invitrogen), and transferred to 0.22 μm nitrocellulose or PVDF membranes. Molecular weights were estimated using Precision Plus Protein Kaleidoscope Prestained Protein Standard (Bio-Rad #1610375). Primary antibodies included rabbit polyclonal anti-BAK aa23-38 (1:5000, Sigma #B5897, RRID:AB_258581), rat monoclonal anti-BAK (clone 4B5, in-house), anti-cytochrome *c* (1:2000, BD Pharmingen #556433, RRID: AB_396417). These two anti-BAK antibodies recognize all BAK after its denaturation on SDS-PAGE or transfer in SDS after BN-PAGE, as they both bind to linear epitopes (aa23-38 and BH3 domain, respectively). Detection was achieved using horseradish peroxidase (HRP)-conjugated anti-rabbit (1:5000, Southern Biotech #4010-05, RRID: AB_2632593), anti-rat (1:5000, Southern Biotech #3010-05, RRID: AB_2795801) and anti-mouse (1:2000, Southern Biotech #1010-05, RRID: AB_2728714) secondary antibodies. To avoid signals from antibody light chains in immunoprecipitation samples, heavy chain-specific HRP-conjugated goat anti-rabbit IgG (1:5000, Southern Biotech #4041-05, RRID: AB_2795946) and goat anti-rat IgG (1:5000, Southern Biotech #3030-05, AB_2716837) were also used. Proteins were visualized by Luminata Forte Western HRP substrate (Millipore #WBLUF0500) on a ChemiDoc TM Touch Imaging System (Bio-Rad Cat#1708370), and images processed with ImageLab Software (Bio-Rad v6.1). Uncropped blots are shown in Supplementary Material File.

### Recombinant protein expression and purification

hBAKΔN22ΔC25Δcys (BAKΔTM) wild-type, BAK mutants (M60G, M60W), avi-tagged BAKΔTM and mBAKΔN20ΔC25Δcys were produced as previously described [40, 47]. Here and elsewhere Δcys means replacement of wild-type cysteine residues with serine. BAK constructs were cloned into the pGEX 6P3 vector and the corresponding plasmids were transformed into BL21 (DE3) *Escherichia coli* cells and grown in Super Broth (induction at OD (600 nm) = 1 with 1 mM IPTG for 3 h at 37 °C). Cells were lyzed in 50 mM Tris pH 8.0, 150 mM NaCl, 1 mM EDTA and 1.5 µg/mL DNase I (Roche, Mannheim, Germany). Supernatants were passed over glutathione resin (GenScript, Piscataway, NJ, USA) and the GST-tag removed by proteolysis using HRV-3C protease (1 ml at 0.2 mg/ml) overnight at 4 °C. BAK proteins were eluted as the flowthrough using lysis buffer without the DNase. All proteins were further purified by size exclusion chromatography (Superdex S75 10/300) in TBS (20 mM Tris pH 8.0, 150 mM NaCl) at 4 °C and snap frozen in liquid nitrogen and stored at −80 °C.

### Preparation of 14G6 Fab-BAK complex for crystallization

Papain (P3125-100 mg, Sigma) was activated by diluting to 1 mg/ml in PBS containing 10 mM cysteine and 20 mM EDTA and incubated on ice for 10 min. Activated papain (final concentration 0.05 mg/ml in PBS) was added to 14G6 (1 mg/ml) and cleaved for 2 h at 37 °C. The reaction was stopped by addition of iodoacetamide to a concentration of 30 mM and incubation on ice for 30 min. The cleaved mixture was dialyzed into 10 mM sodium acetate, pH 4.5 and purified by cation exchange on a Mono S 5/50 column at 2 ml/min 0–100% Buffer B (10 mM sodium acetate, 500 mM NaCl) over 20 column volumes. 1 M Tris pH 8 was added to fractions containing Fab. Purified 14G6 Fab was mixed with a 1.5-fold excess of BAKΔTM and purified on a Superdex 200 Increase 10/300 column (Cytiva) in TBS buffer. The complex was concentrated to 5.4 mg/ml, flash frozen and stored at −80 °C until use.

### Crystallization and structure determination

Crystals were obtained by sitting drop vapour diffusion with a reservoir containing 0.09 M Bis-tris chloride pH 5.5, 22.5% PEG 3350, 4% acetonitrile. X-ray diffraction data were collected at the Australian Synchrotron MX2 Beamline using an Eiger 16 M detector [64]. Data were processed using XDS [65]. The structure was determined by molecular replacement using MolRep [66] with 2YV6 [67] (BAK) and 5W5Z [39] (Fab) as the search models. The structure was refined in Refmac5 [68] and Phenix [69], with iterative model building in COOT [70].

### Surface Plasmon Resonance

Surface Plasmon Resonance (SPR) data were collected on a Biacore S200 (Cytiva). 14G6 was diluted to 2 µg/mL in running buffer and approximately 150 Resonance Units (RU) captured on a Protein A chip (Cytiva). Kinetics for each BAK construct were determined using a single-cycle kinetic method, with injection time 300 sec and a final dissociation time of 2400 sec. All curves were reference and blank subtracted and analyzed with Biacore S200 Evaluation software using 1:1 binding kinetic model. All experiments were carried out at 25 °C using DPBS + 0.005% Tween-20 as running buffer. Kinetic and affinity parameters are quoted as the average ± standard deviation of three independent experiments.

### BID binding to BAK

BID R84C BH3 peptide (Biotin-DSESQEDIICNIARHLAQVGDSMDRSIPPGLVNGL-NH_2_) was synthesized by Mimotopes and stored as a 2 mM stock in DMSO. 9 µM BAKΔcysΔN22ΔC25 CT His with K113C mutation was pre-incubated with 1.5× excess of 14G6 antibody before addition of 4× excess of BID R84C BH3 peptide and treatment with a final concentration of 2 mM copper (II) 1,10-phenanthroline (CuPhe). The reaction was incubated for 1 h at room temperature and stopped by addition of EDTA to 20 mM. Samples were then run on non-reducing SDS-PAGE (Bolt Bis-Tris Plus 4–12% (Invitrogen, NW04120BOX) using MES running buffer).

### Supplementary information

Supplementary Data

Supplementary original blots

## Data Availability

The coordinates for BAK-14G6 have been deposited in the PDB with accession code 8UKY. All other data are available from the corresponding authors upon request.
