# Supplementary material for: A novel inhibitory BAK antibody enables assessment of non-activated BAK in cancer cells
Source: Cell Death Differ. 2024 Apr 6;31(6):711–21. doi: 10.1038/s41418-024-01289-3 (PMC11164899; doi:10.1038/s41418-024-01289-3)
Supplement: Supplementary file 1 — Supplementary Data [file 41418_2024_1289_MOESM1_ESM.docx]

**SUPPLEMENTARY INFORMATION**

**A novel inhibitory BAK antibody enables assessment of non-activated BAK in cancer cells**

Hema Preethi Subas Satish^1,2,3#^, Sweta Iyer^1,2#^, Melissa X. Shi^1,2^, Agnes W. Wong^1,2^, Karla C. Fischer^1,2^, Ahmad Z. Wardak^1,2^, Daisy Lio^1,2^, Jason M. Brouwer^1,2^, Rachel T. Uren^1,2^, Peter E. Czabotar^1,2^, Michelle S. Miller^1,2^*, Ruth M. Kluck^1,2^*

^1^The Walter and Eliza Hall Institute, Melbourne, VIC 3052, Australia.

^2^Department of Medical Biology, The University of Melbourne, Parkville, VIC 3010, Australia

^3^current affiliation: Vanderbilt Vaccine Center, Vanderbilt University Medical Center, Nashville, TN 37232, USA

^#^these authors contributed equally to this work

**SUPPLEMENTARY METHODS**

**Mouse B cell cloning**

To identify antibodies that may regulate BAK, mouse B cell cloning ^1, 2, 3^ was performed in collaboration with the WEHI Antibody Facility and the WEHI Biologics Initiative. Four mice were immunized with BAKΔTM and splenocytes stored in liquid nitrogen. To select B cells that recognized BAK, splenocytes were thawed and first enriched for B cells by magnetic-activated cell sorting (MACS) using a MACS mouse B cell isolation kit (130-090-862; Miltenyi Biotec). Enriched splenocytes were then incubated (4 °C; in the dark; 35 min) with a mixture of fluorophore-conjugated antibodies ^1^ to select for mouse memory B cells, together with streptavidin tetramers of BAKΔTM to select for cells that bound to BAK. (To generate the streptavidin tetramers of BAKΔTM, avi-tagged BAKΔTM had been biotinylated using BirA enzyme, followed by incubation with either brilliant violet (BV) 510- or BV786-conjugated streptavidin (BioLegend; 405234/405249) at a molar ratio of 1:0.34.) Flow cytometry using BD FACSAria^TM^ III then sorted for BAK-specific IgG-expressing memory B cells (BAK^+^ B220^+^ CD38^+^ IgM^-^ IgD^-^ CD3^-^ F4/80^-^ Gr-1^-^ CD95^-^) ^1^.

To express antibodies, sorted B cells were lyzed, followed by reverse transcription of total RNA and Sanger sequencing of antibody variable heavy (V_H_) and light chains (V_L_) ^3^. For transfection and expression of antibodies, productive antibody heavy and light chain pairs were selected and the V_H_ and V_L_ genes cloned into AbVec-hIgG1 and AbVec-IgKappa vectors which contain human constant regions. Following antibody purification, twelve antibodies bound to BAKΔTM on ELISA. One clone, 14G6, also bound to non-activated full-length BAK when tested by both immunoprecipitation and gel-shift on BN-PAGE (**Figure 1**).

**Large scale expression and purification of 14G6**

To utilize 14G6 in the analysis of BAK changes during apoptosis, large scale expression was performed in Expi293F cells. Cells were transfected with equal amounts of AbVec-IgH and AbVec-IgKappa plasmids cloned with 14G6 V_H_ or V_L_. The plasmids were mixed with LPEI at a molar ratio of 1:3, then added dropwise into cells shaking at 140 rpm, and incubated at 37 °C. To enhance antibody production, valproic acid (2.5 mM) and glucose (6 gm/l) were added on days 1 and 2 after transfection, respectively. On day 6, supernatant was harvested (4,000 *g*) and buffer exchanged by dialysis into Dubelco’s phosphate buffered saline (DPBS) overnight at 4 °C, filtered (0.22 μm), and loaded onto a HiTrap Protein A antibody purification column (17040201; Cytiva) using a FPLC system (Bio-Rad). The antibodies were eluted with 0.1 M citric acid, pH 3.0, and each 1 ml fraction neutralized with 300 μl 1 M Tris, pH 9. Relevant fractions (based on chromatogram) were pooled and buffer exchanged into DPBS and concentrated using Amicon^®^ Ultra-15 centrifugal filter units with MWCO of 10 kDa (Millipore Sigma). The antibodies were further purified by size exclusion chromatography using Superdex 200 10/300 GL column (17517501; Cytiva) in DPBS, and stored at -20 °C.

**Detection of BAK and cytochrome *c* by flow cytometry**

To measure non-activated and activated BAK in the same cells, digitonin-permeabilized MEFs treated with or without 100 nM cBID were resuspended in Fixation buffer (Invitrogen/eBiosciences #00-5523) at 4 °C for 30-60 min prior to two washes with Permeabilization buffer (Invitrogen/eBiosciences #00-5523). Cells were then stained by resuspension in Permeabilization buffer containing 14G6 (1:800) or antibody to activated BAK (1:100, mouse monoclonal clone G317‑2, BD Biosciences #556382) and incubated at 4 °C for 30 min. Cells were washed twice with Permeabilization buffer and stained at 4 °C for 30 min with goat anti-human AF647 (1:200, Invitrogen #A-21445) and goat anti-mouse Pacific Blue (1:200, ThermoFisher #P31582, AB_10374586). Stained cells were washed twice in Permeabilization buffer and analyzed on a FACS Fortessa 1 flow cytometer (BD Biosciences, San Jose, CA, USA; BD FACS Diva software).

To measure activated BAK and cytochrome *c* release in the same cells, digitonin-permeabilized cells were incubated with cBID, 7D10 or heat, and cells stained by resuspension in Permeabilization buffer containing G317‑2 (1:1,600) and incubated for 30 min at 4 °C. Cells were washed twice with Permeabilization buffer and stained for 30 min at 4 °C with goat anti-mouse Pacific Blue together with anti-cytochrome *c*-APC (1:50, clone REA702, Miltenyi Biotec #130-111-368).

**Detection of non-activated BAK by immunocytochemistry**

*bak^-/-^bax^-/-^* MEFs expressing hBAK were cultured in 8 well chamber slides and first incubated with 0.25 μM MitoTracker^TM^ Deep Red FM (Invitrogen #M22426) at 37 °C for 1 h to stain mitochondria. Cells were then permeabilized with 0.025 % digitonin at RT for 15 min, washed and incubated with 100 nM cBID at 30 °C for 35 min. To visualise non-activated hBAK, samples were fixed with Fixation buffer (Invitrogen/eBiosciences #00-5523) at 4 °C for 35 min and incubated with Alexa Fluor-488 conjugated 14G6 antibody (1:500, in house) at RT for 2 h. To stain nuclei, Hoescht (1 μg/ml, Thermo Scientific #62249) was added to the final wash. Images were acquired using a Zeiss confocal microscope LSM880 with a 63X oil objective lens and processed using Image J software (v2.14.0).

**SUPPLEMENTARY FIGURES**

**
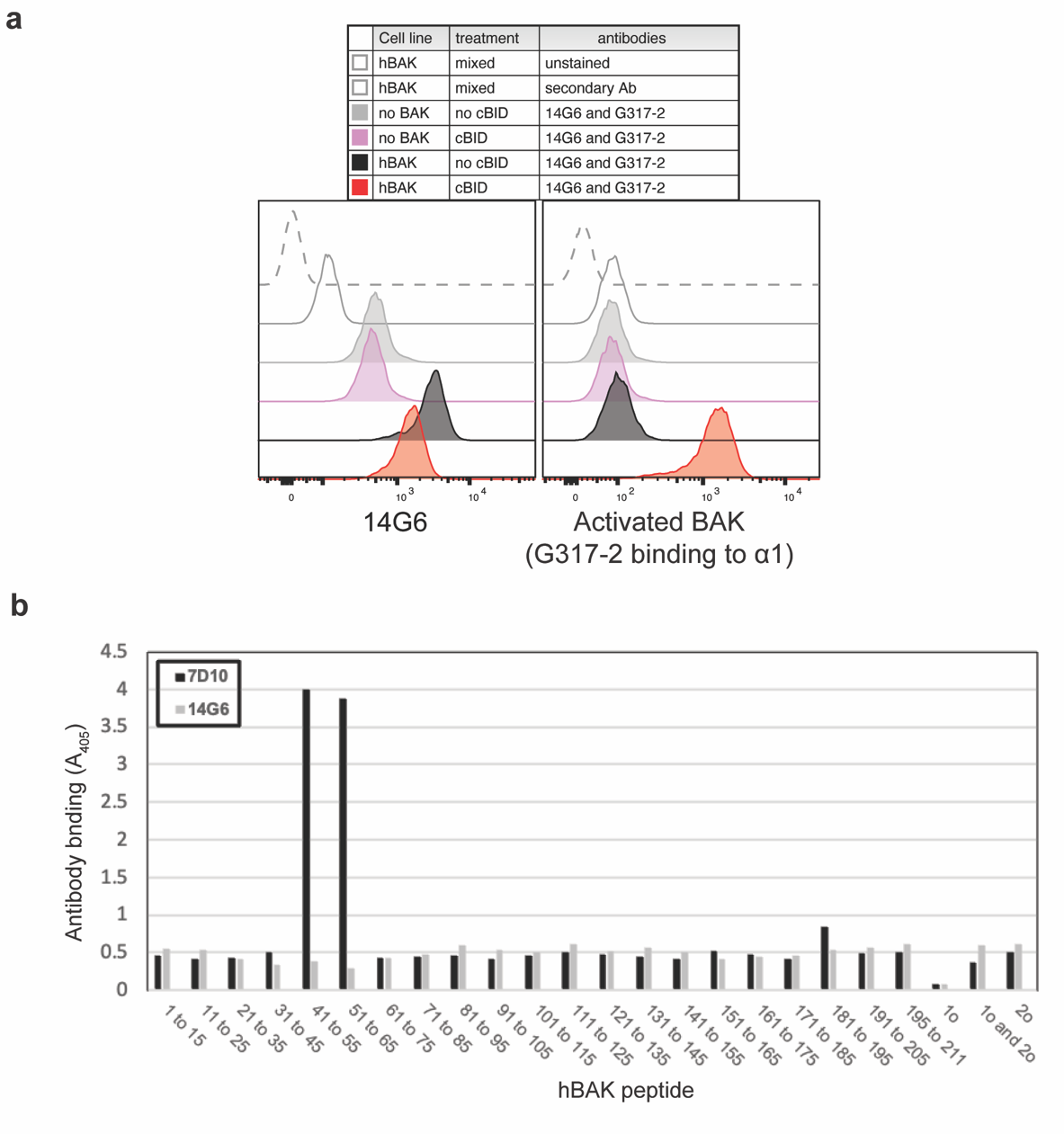
**

**Figure S1. 14G6 binding to non-activated BAK.**

**a.** 14G6 specificity for non-activated BAK on flow cytometry. Membrane fractions from *bak^-/-^bax^-/-^* MEFs (no BAK) or *bak^-/-^bax^-/-^* MEFs expressing hBAK (hBAK) were incubated with or without cBID (30 ℃, 30 min). Membrane fractions were fixed and left unstained or stained as indicated with 14G6 and G317-2 and related secondary antibodies prior to analysis by flow cytometry. Data are representative of two independent experiments.

**b.** 14G6 does not bind any BAK peptide on peptide scanning array. Peptides (15-mer, five residue overlap) encompassing the full length of human BAK protein were coated onto streptavidin-coated plates and incubated with either 7D10 or 14G6, and bound antibody detected by binding of HRP-conjugated secondary antibodies, as described previously ^4^. Data is representative of two independent experiments.

**Figure S2. 7D10 will bind BAK already bound to 14G6**

Membrane fractions from Figure 2a were run on BN-PAGE and blotted for BAK (clone aa23-38) as in Figure 1c. Experiment is representative of three independent experiments.

**
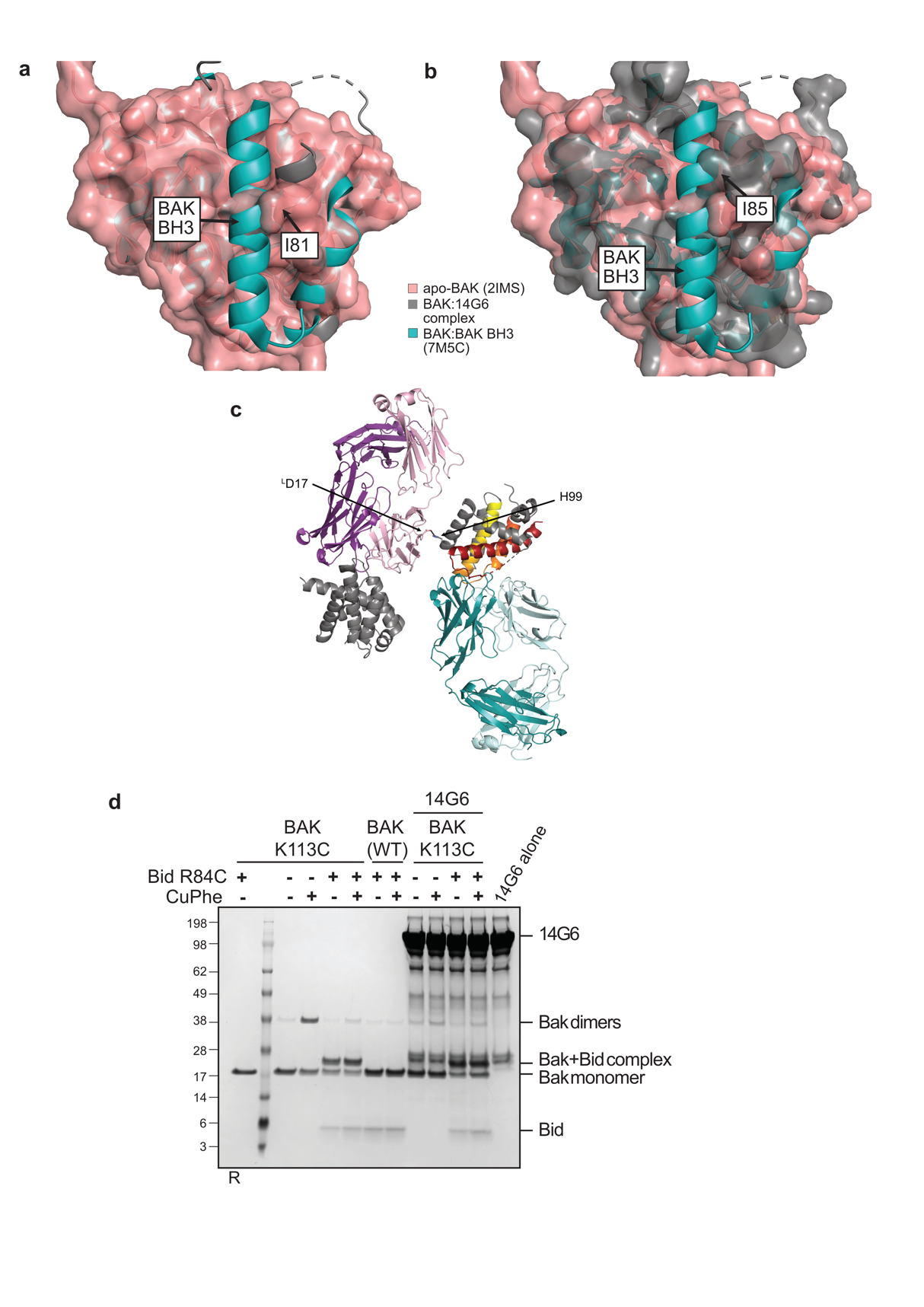
**

**Figure S3. 14G6 bound BAK has a more closed groove but can still bind BID**

**a.** Structural alignment of apo-BAK (2IMS, pink surface), 14G6-bound BAK (grey cartoon) and BAK-BH3 from 7M5C to highlight BH3-peptide binding to the groove.

**b.** As for (a) but with 14G6-bound BAK shown as grey surface to highlight the more closed groove. I85 points into the groove and creates a clash with potential BH3 binding.

**c.** The crystal has two copies of the 14G6 Fab:BAK complex. There is a hydrogen bond between D:BAK H99 and L:D17 from the light chain in the other complex. This may affect the conformation of α3.

**d.** BID BH3 peptide can still be linked to groove of 14G6-bound BAK. Wild type BAKΔTM or the K113C mutant were incubated with the BID R84C BH3 peptide and incubated with the oxidant CuPhe. Disulphide linkage of the BAK+BID complex represents binding, and still occurs if 14G6 was pre-incubated with BAK. Coomassie-stained SDS-PAGE. Data is representative of three independent experiments. R – this lane was run under reducing conditions.

**
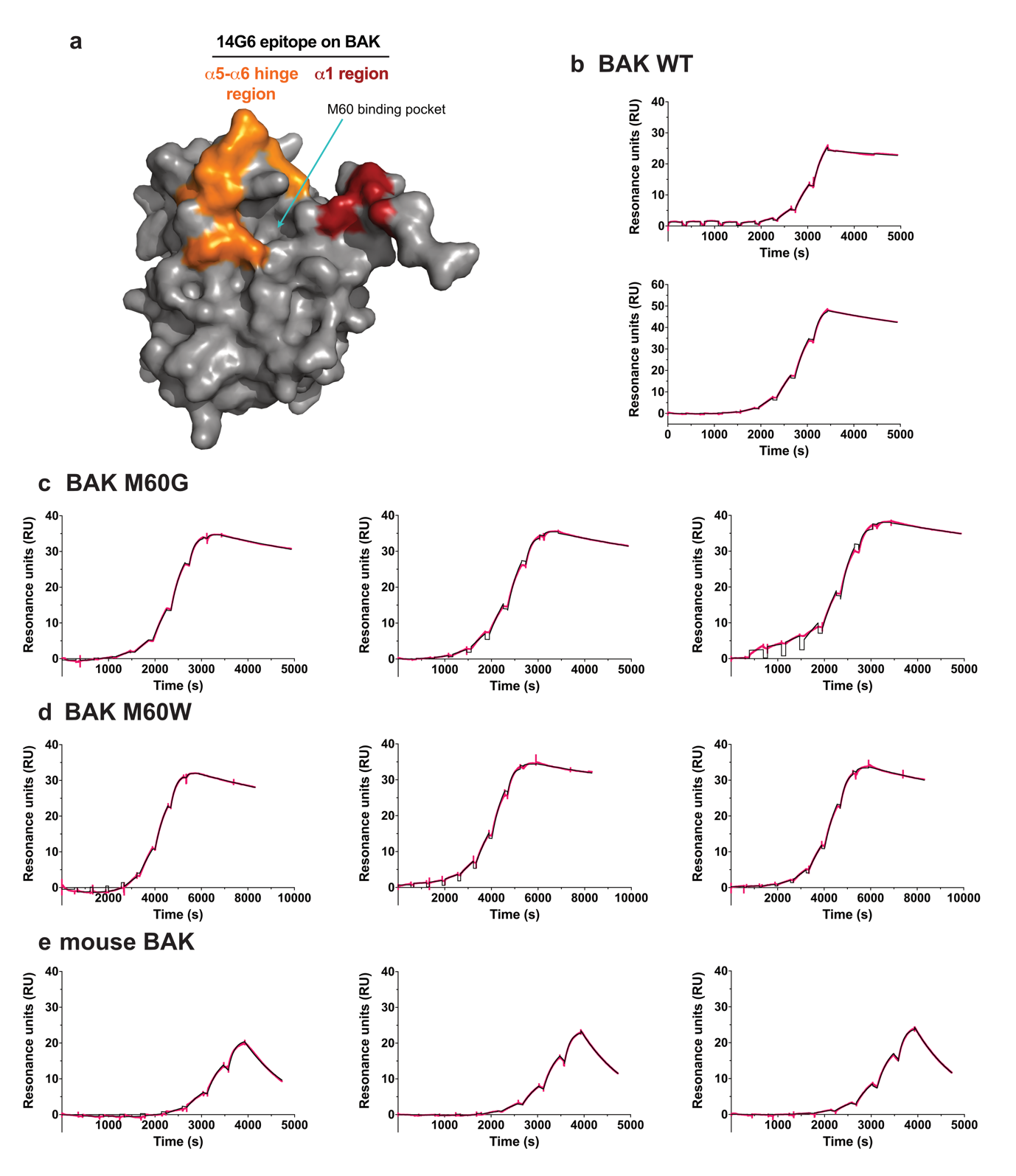
**

**Figure S4. Mutagenesis of 14G6 epitope in BAK alters binding affinity**

**a.** Surface model of BAK highlighting 14G6 epitope and relevant mutations.

**b-e.** 14G6 SPR binding curves (related to Table 1). Single cycle kinetics were performed with 14G6 captured on a Protein A chip and increasing concentrations of BAKΔTM flowed over the chip. Buffer and eight concentrations from a threefold dilution series were used for each run with the following top concentrations: WT (1 µM), M60G (300 nM), M60W (3 µM), mBAK (10 µM).

**
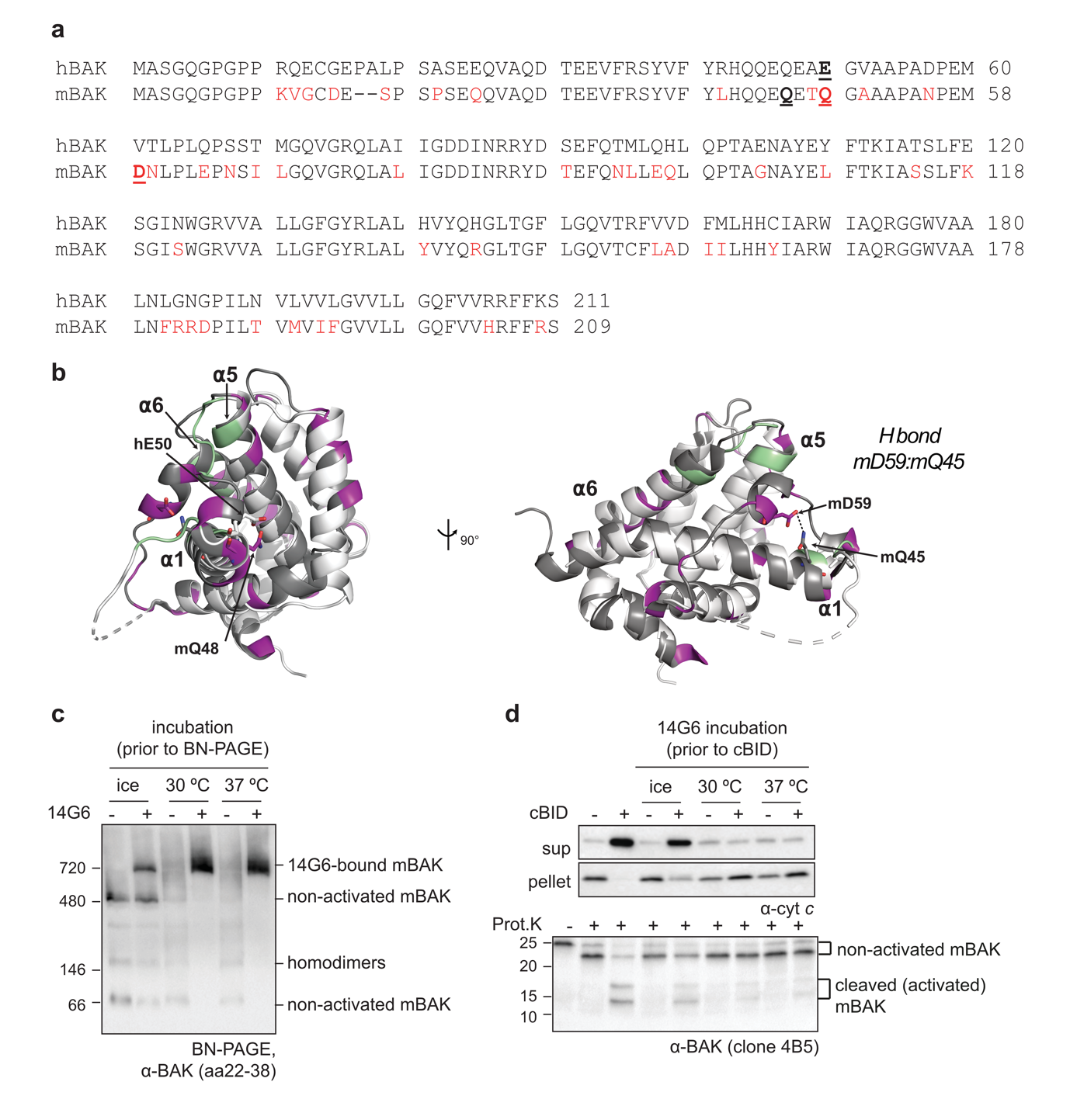
**

**Figure S5. 14G6 binding and inhibition of mouse BAK**

**a.** Sequence alignment of human and mouse BAK. Non-conserved residue positions are shown in red. Residues involved in hydrogen bonds in (b) are in bold.

**b.** Structural alignment of mouse (6MCY, dark grey) and human BAK (this paper, white). Non-conserved residue positions are shown in purple. The contact surface with 14G6 is shown in light green.

**c.** 14G6 binding to mouse BAK in mitochondria is efficient at 30 °C. Permeabilized *bax^-/-^* MEFs were incubated with 14G6 at the indicated temperatures for 30 min, then solubilized with 1% digitonin, run on BN-PAGE and immunoblotted for BAK (aa23-38) as in Figure 1c. Data are representative of two independent experiments.

**d.** 14G6 pre-incubation at 30 °C inhibits activation of mouse BAK and cytochrome *c* release. Permeabilized *bax^-/-^* MEFs were incubated with 14G6 at the indicated temperatures for 30 min prior to incubation with cBID (30 ℃, 30 min). Aliquots were separated to supernatant and pellet fractions and immunoblotted for cytochrome *c* (top panel), or were incubated with proteinase K (Prot. K) and immunoblotted with antibody to the BAK BH3 domain (clone 4B5; lower panel). Data is representative of two independent experiments.

**Figure S6. BAK and BAX activation by BH3 mimetic treatment demonstrated by increased cleavage by proteinase K**

Four acute myeloid leukemia cell lines were incubated with 1 µM iBCL2 (venetoclax), iMCL1 (S63845), iBCLxL (A-1331852), alone or in combination for 3 h, as in Figure 4c. Cell lysates were incubated with proteinase K and blotted for BAK (as in Figure 2b). Data is representative of at least two independent experiments.

**SUPPLEMENTARY REFERENCES**

1. Carbonetti S, Oliver BG, Vigdorovich V, Dambrauskas N, Sack B, Bergl E*, et al.* A method for the isolation and characterization of functional murine monoclonal antibodies by single B cell cloning. *J Immunol Methods* 2017, **448:** 66-73.

2. Starkie DO, Compson JE, Rapecki S, Lightwood DJ. Generation of Recombinant Monoclonal Antibodies from Immunised Mice and Rabbits via Flow Cytometry and Sorting of Antigen-Specific IgG+ Memory B Cells. *PLoS One* 2016, **11**(3)**:** e0152282.

3. von Boehmer L, Liu C, Ackerman S, Gitlin AD, Wang Q, Gazumyan A*, et al.* Sequencing and cloning of antigen-specific antibodies from mouse memory B cells. *Nat Protoc* 2016, **11**(10)**:** 1908-1923.

4. Alsop AE, Fennell SC, Bartolo RC, Tan IKL, Dewson G, Kluck RM. Dissociation of Bak α 1 helix from the core and latch domains is required for apoptosis. *Nature Communications* 2015, **6**.
