# Supplementary material for: A novel inhibitory BAK antibody enables assessment of non-activated BAK in cancer cells
Source: Cell Death Differ. 2024 Apr 6;31(6):711–21. doi: 10.1038/s41418-024-01289-3 (PMC11164899; doi:10.1038/s41418-024-01289-3)

Figure 1c

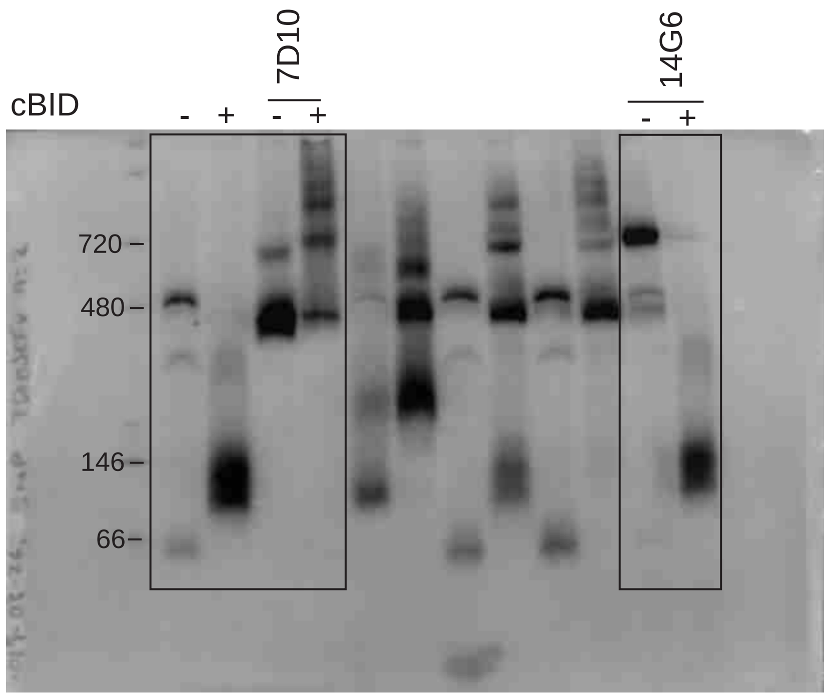

BN-PAGE, α-BAK (aa22-38)

Figure 1b

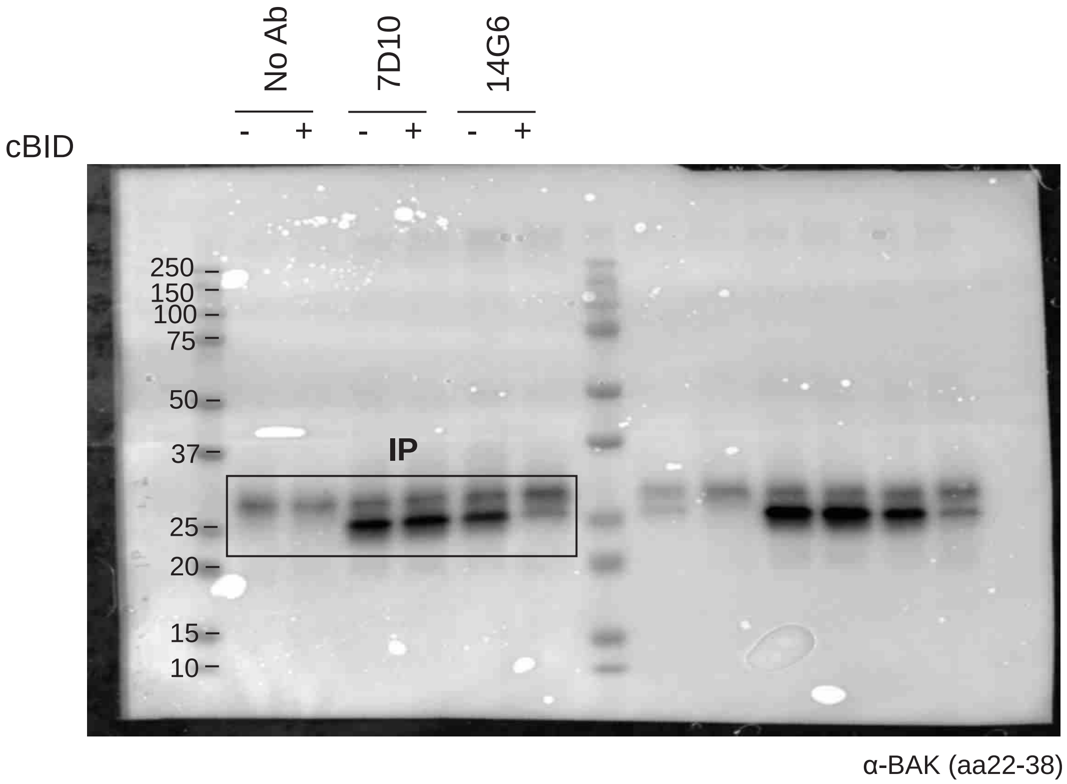
No Ab
7D10
14G6
cBID
- + - + - +
250
150
100
75
50
37
25
20
15
10
IP
α-BAK (aa22-38)

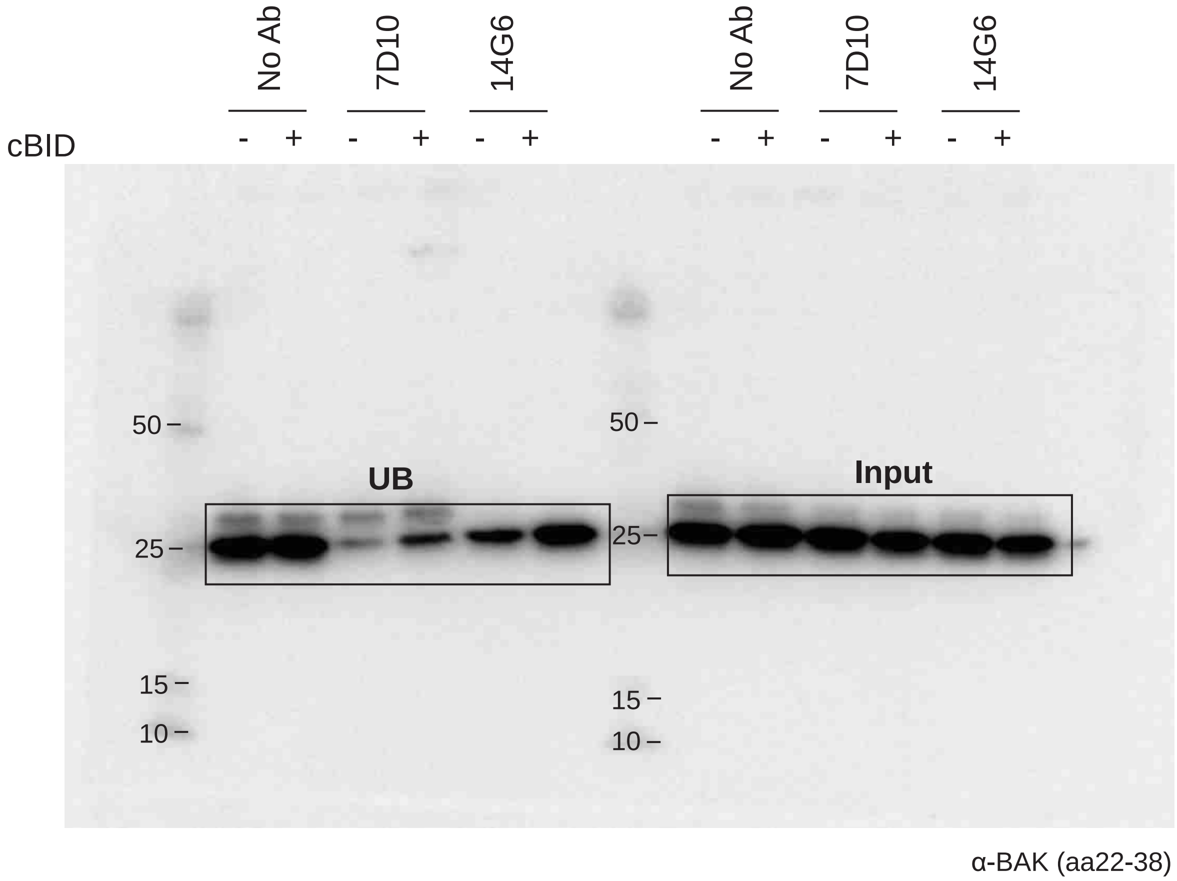
No Ab
7D10
14G6
No Ab
7D10
14G6
cBID
- + - + - + - + - + - +
50
25
15
10
UB
50
25
15
10
Input
α-BAK (aa22-38)

Figure 2a, b

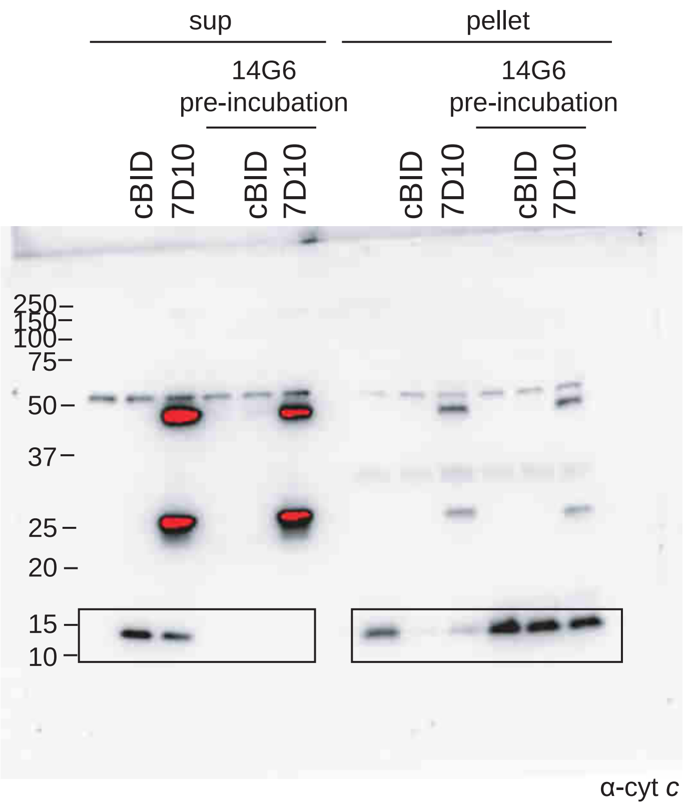
sup
pellet
14G6
pre-incubation
14G6
pre-incubation
cBID
7D10
cBID
7D10
cBID
7D10
cBID
7D10
250
150
100
75
50
37
25
20
15
10
α-cyt c

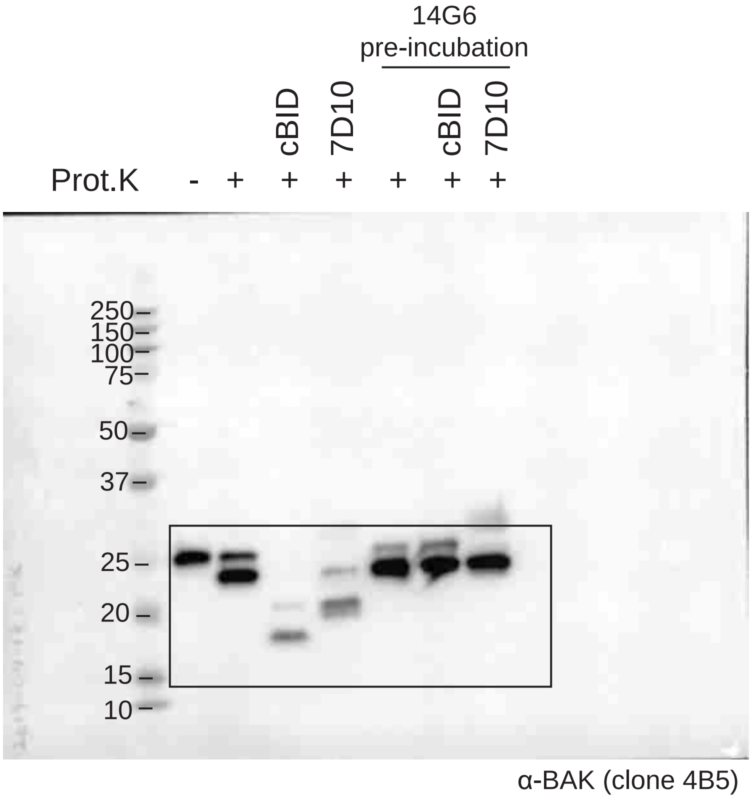
14G6
pre-incubation
cBID
7D10
cBID
7D10
Prot.K - + + + + + +
250
150
100
75
50
37
25
20
15
10
α-BAK (clone 4B5)

Figure 4a

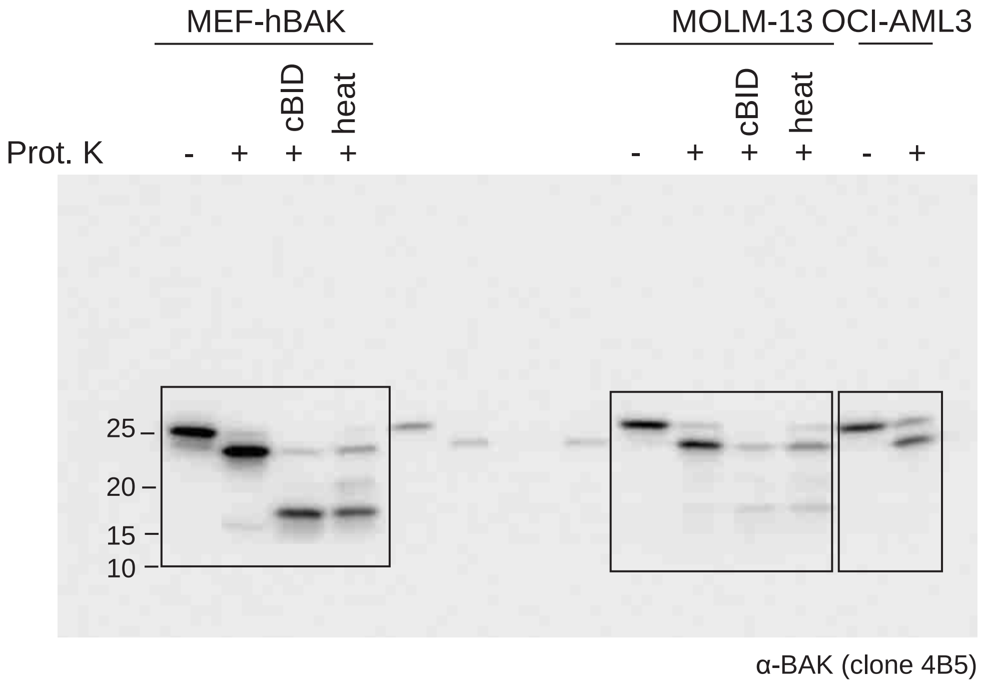

MEF-hBAK
MOLM-13
OCI-AML3
cBID
heat
cBID
heat
Prot. K
- + + +
- + + +
- +
25
20
15
10
α-BAK (clone 4B5)

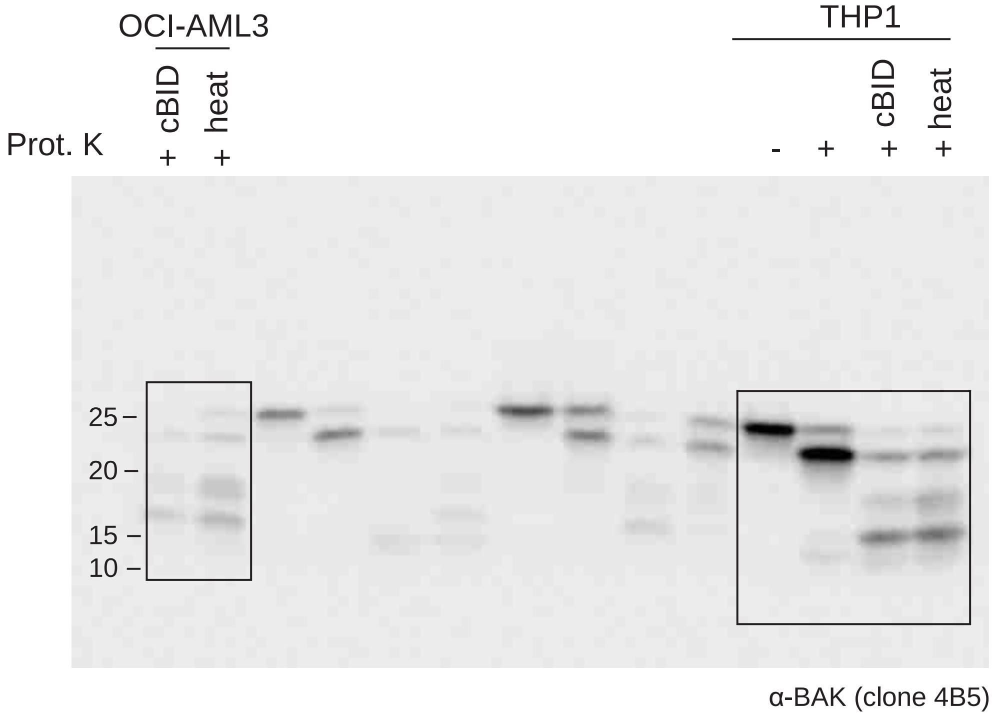

OCI-AML3
THP1
cBID
heat
cBID
heat
Prot. K
+ +
- + + +
25
20
15
10
α-BAK (clone 4B5)

Figure 4a Cont'd

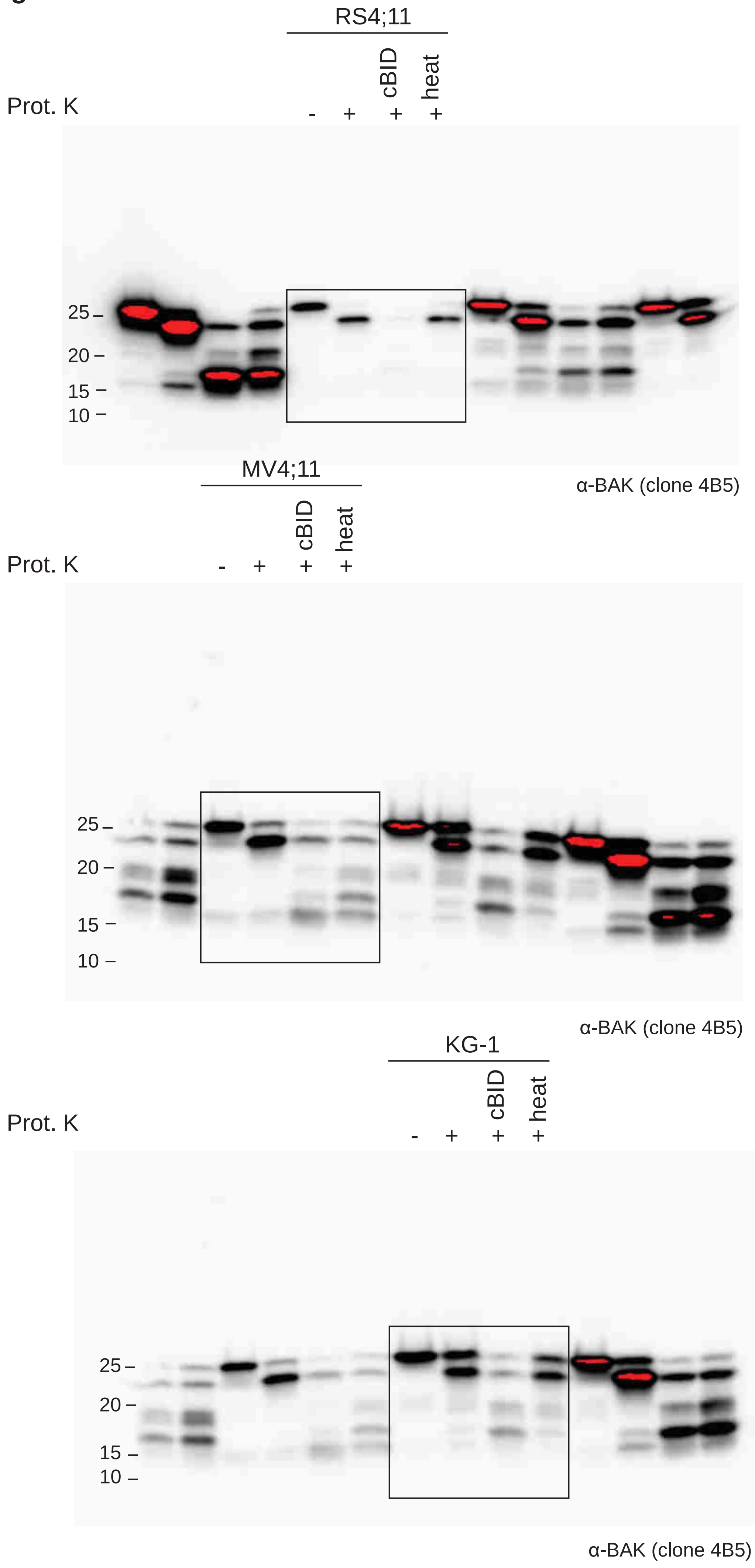

Figure 4b

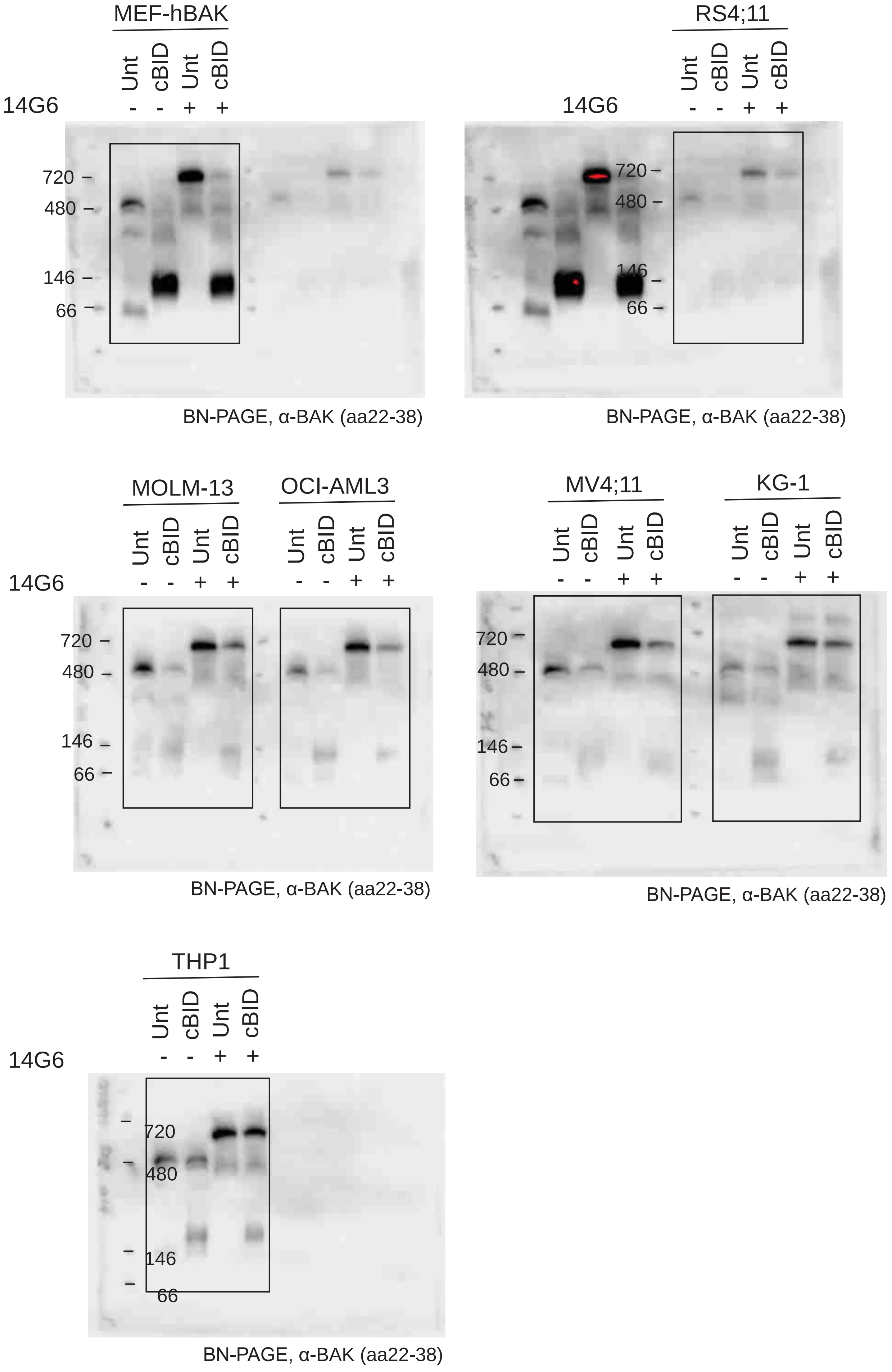

MEF-hBAK
Unt cBID Unt cBID
14G6 - - + +
720
480
146
66
BN-PAGE, α-BAK (aa22-38)
RS4;11
Unt cBID Unt cBID
14G6 - - + +
720
480
146
66
BN-PAGE, α-BAK (aa22-38)
MOLM-13
OCI-AML3
Unt cBID Unt cBID
14G6 - - + +
720
480
146
66
BN-PAGE, α-BAK (aa22-38)
MV4;11
KG-1
Unt cBID Unt cBID
- - + +
720
480
146
66
BN-PAGE, α-BAK (aa22-38)
THP1
Unt cBID Unt cBID
14G6 - - + +
720
480
146
66
BN-PAGE, α-BAK (aa22-38)

Figure 4c

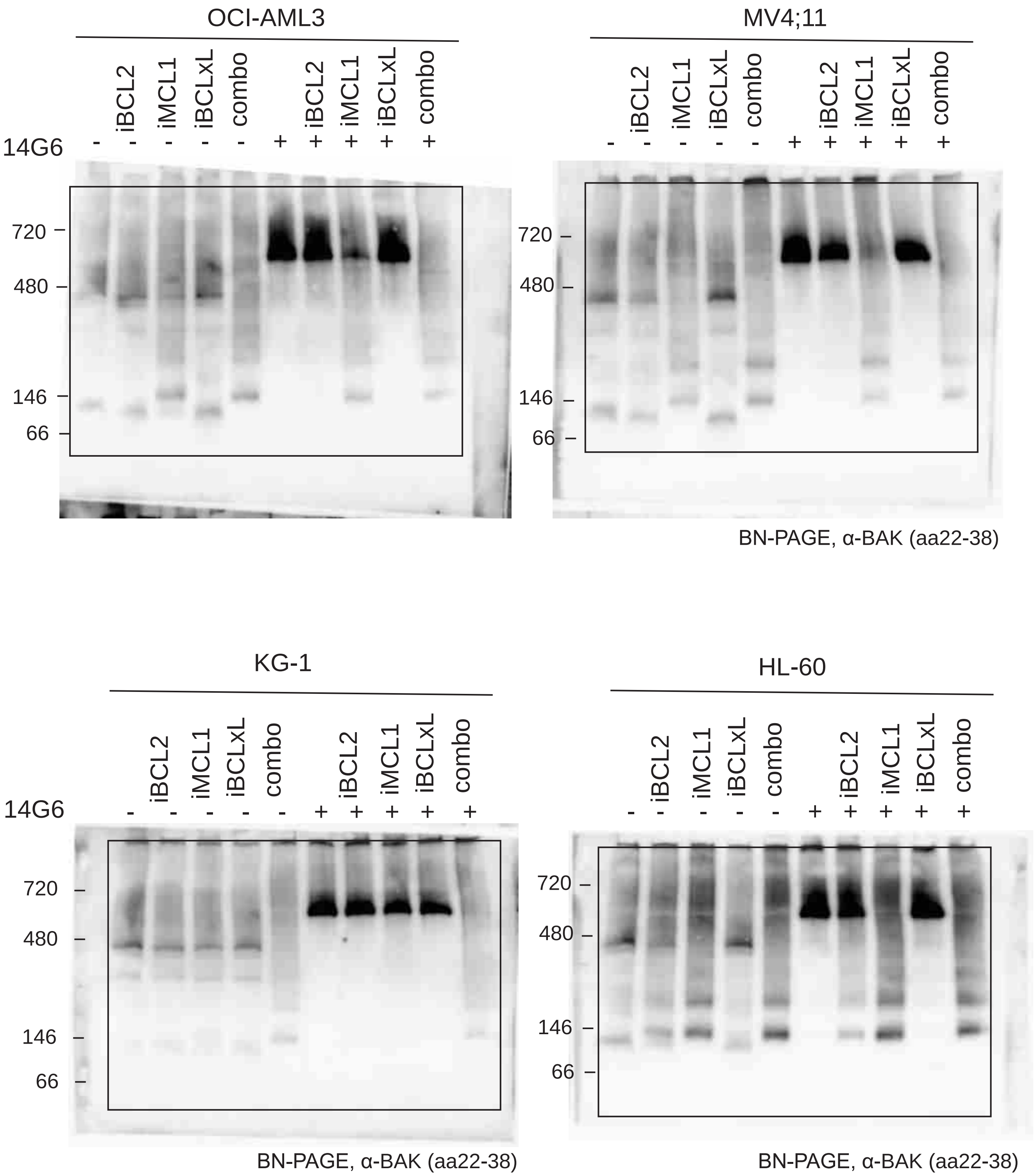
OCI-AML3
MV4;11
KG-1
HL-60
14G6
iBCL2
iMCL1
iBCLxL
combo
720
480
146
66
BN-PAGE, α-BAK (aa22-38)

Figure S2

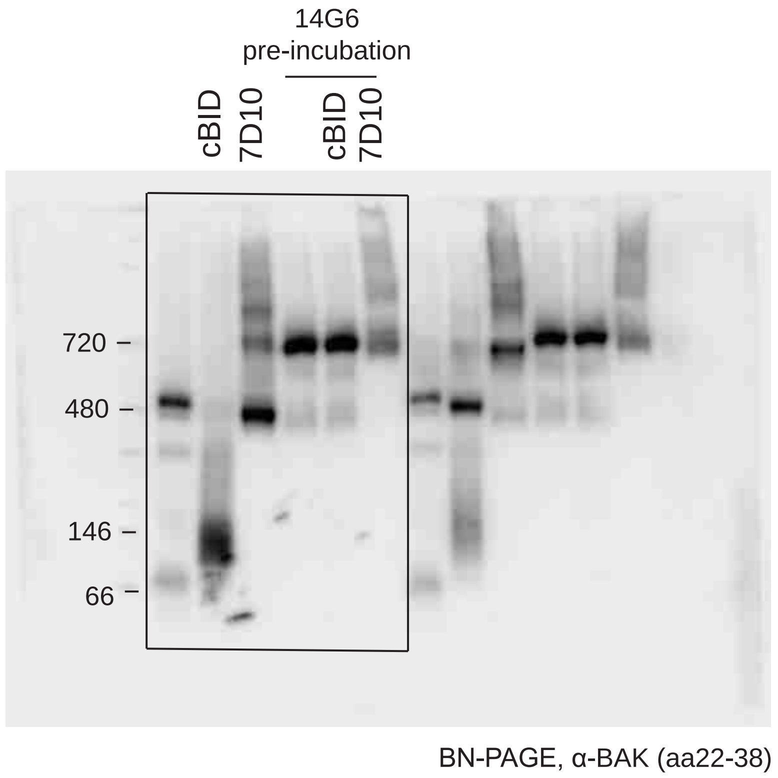
14G6
pre-incubation
cBID
7D10
cBID
7D10
720
480
146
66
BN-PAGE, α-BAK (aa22-38)

Figure S3d

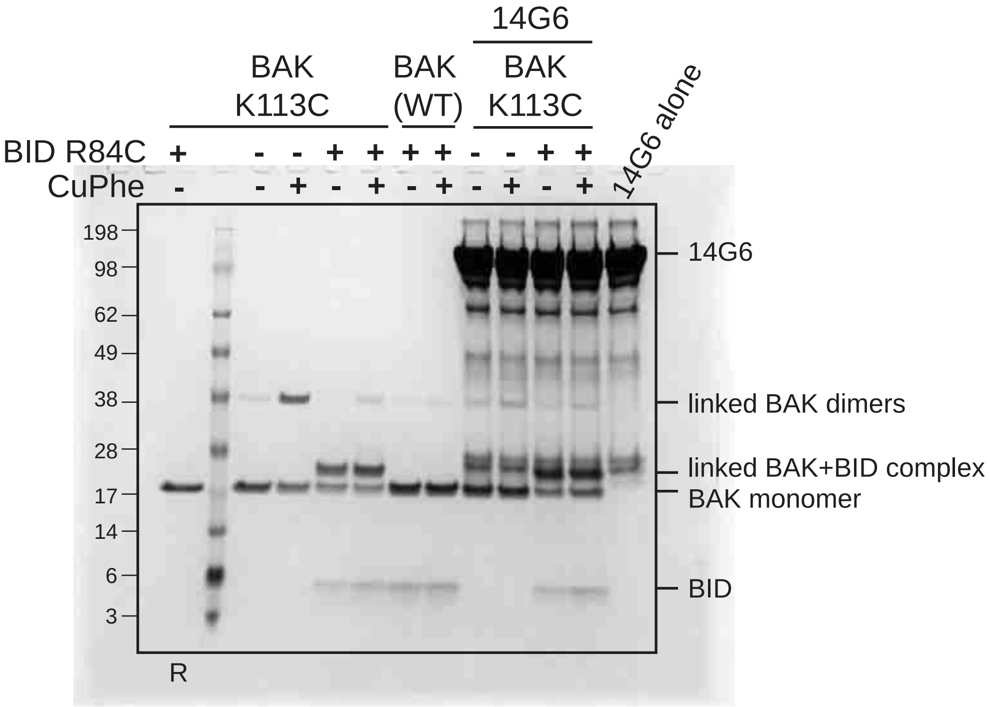

14G6
BAK K113C
BAK (WT)
BAK K113C
14G6 alone
BID R84C
CuPhe
+ - - + + + + - - + +
- - + - + - + - + - +
198
98
62
49
38
28
17
14
6
3
R
14G6
linked BAK dimers
linked BAK+BID complex
BAK monomer
BID

Figure S5c

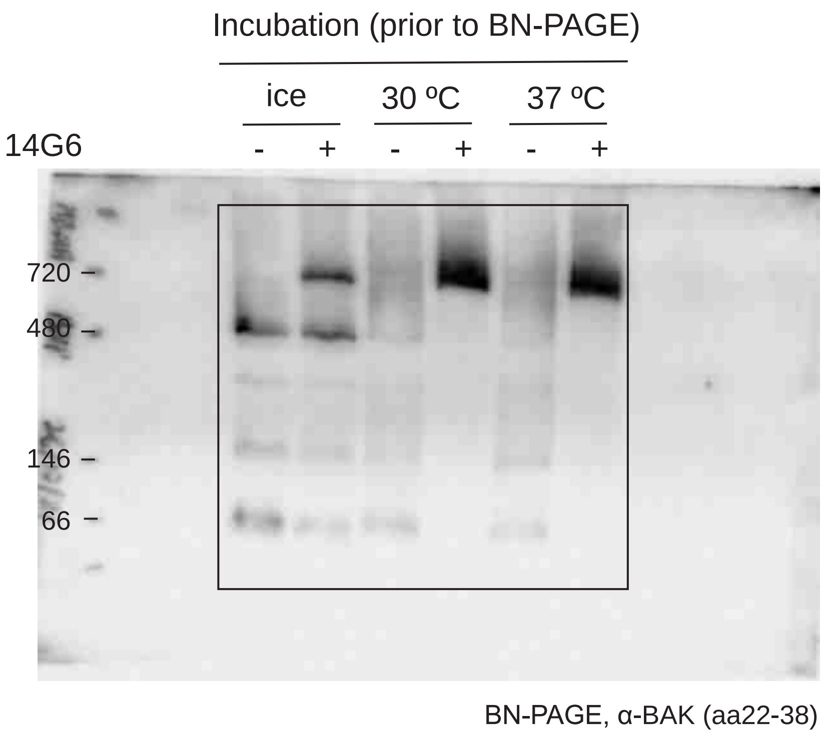

Incubation (prior to BN-PAGE)
ice
30 ºC
37 ºC
14G6
-
+
-
+
-
+
720
480
146
66
BN-PAGE, α-BAK (aa22-38)

Figure S5d

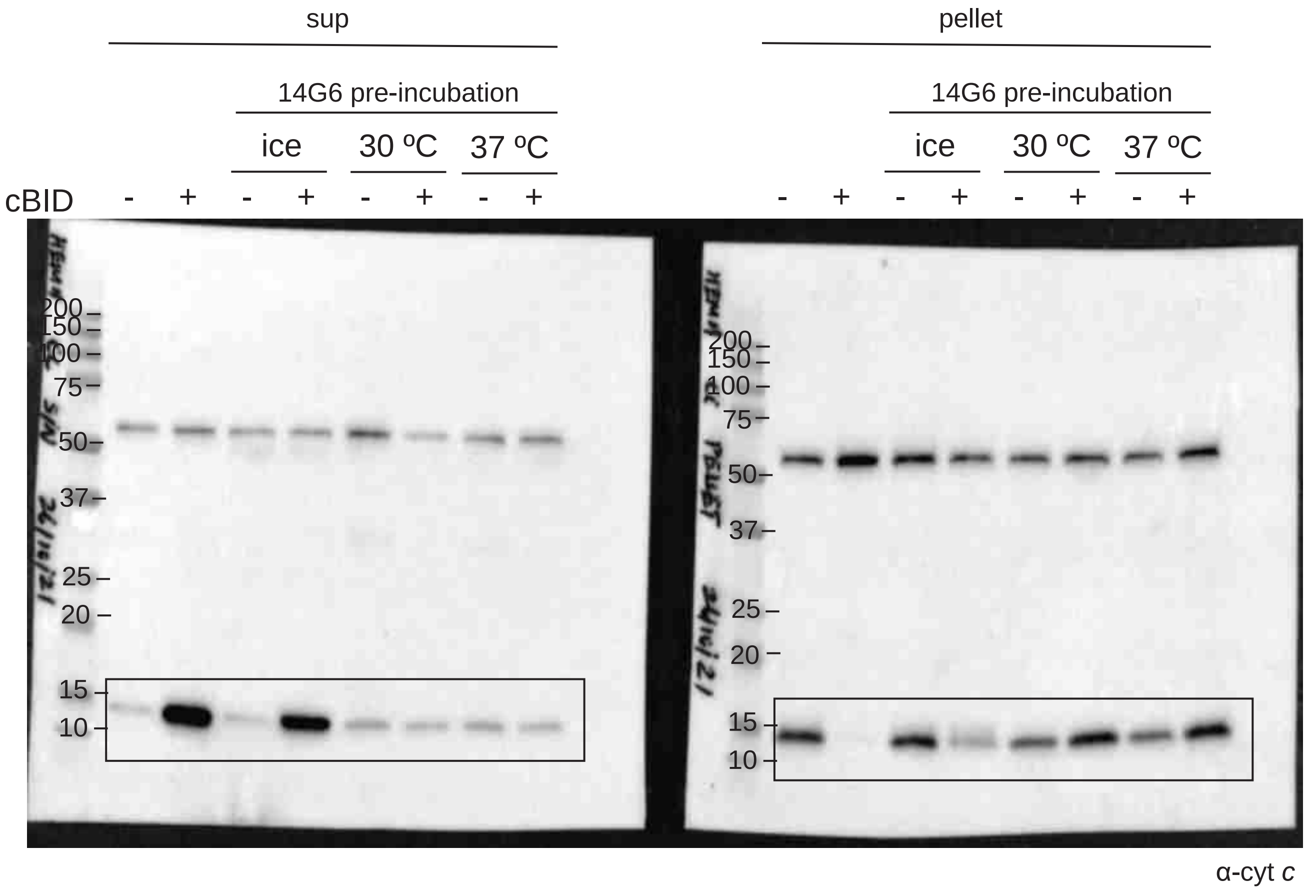
sup
pellet
14G6 pre-incubation
14G6 pre-incubation
ice
30 ºC
37 ºC
ice
30 ºC
37 ºC
cBID - + - + - + - +
- + - + - + - +
200
150
100
75
50
37
25
20
15
10
200
150
100
75
50
37
25
20
15
10
α-cyt c

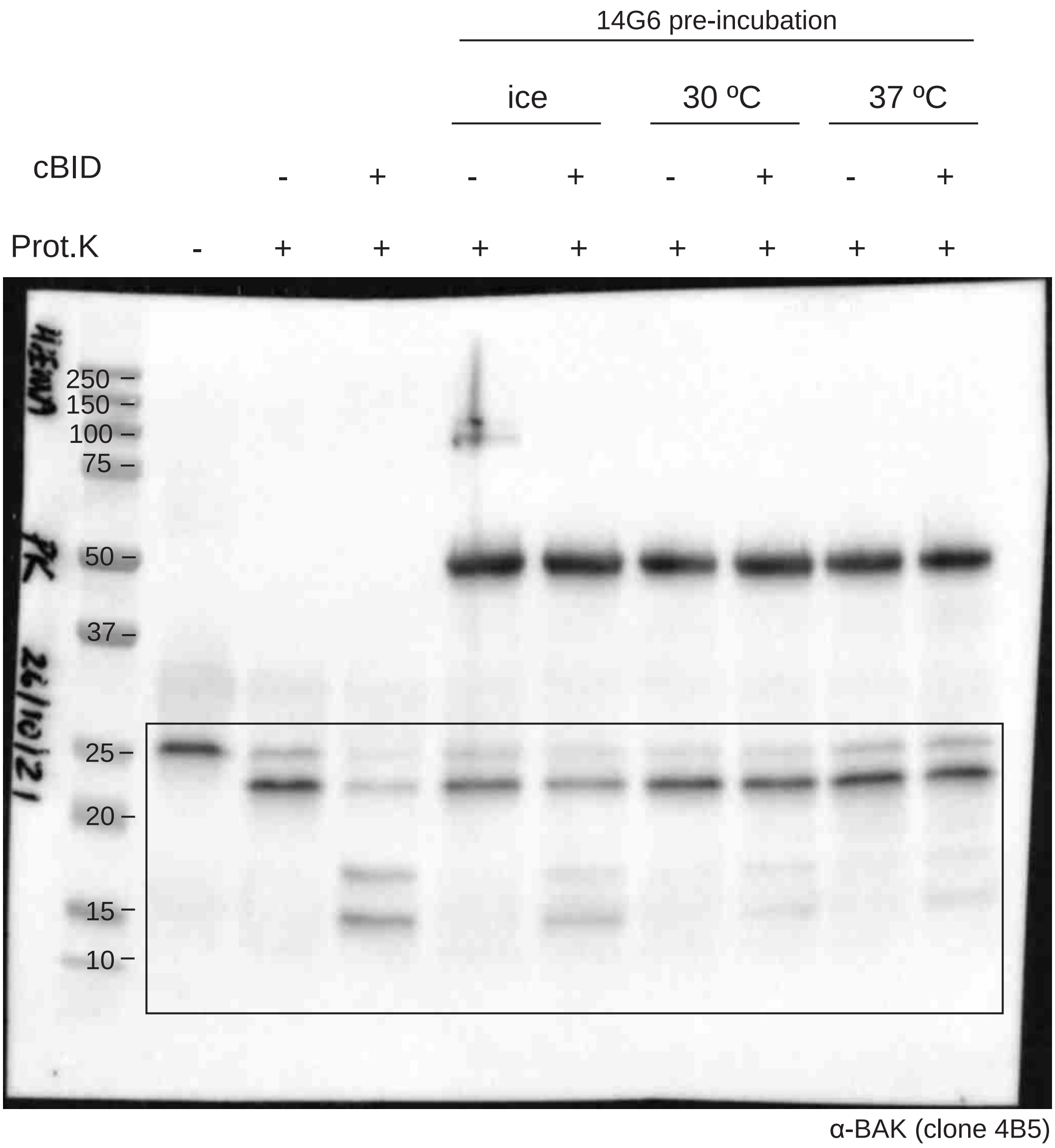
14G6 pre-incubation
ice
30 ºC
37 ºC
cBID - + - + - + - +
Prot.K - + + + + + + + +
250
150
100
75
50
37
25
20
15
10
α-BAK (clone 4B5)

Figure S6 (BAK)

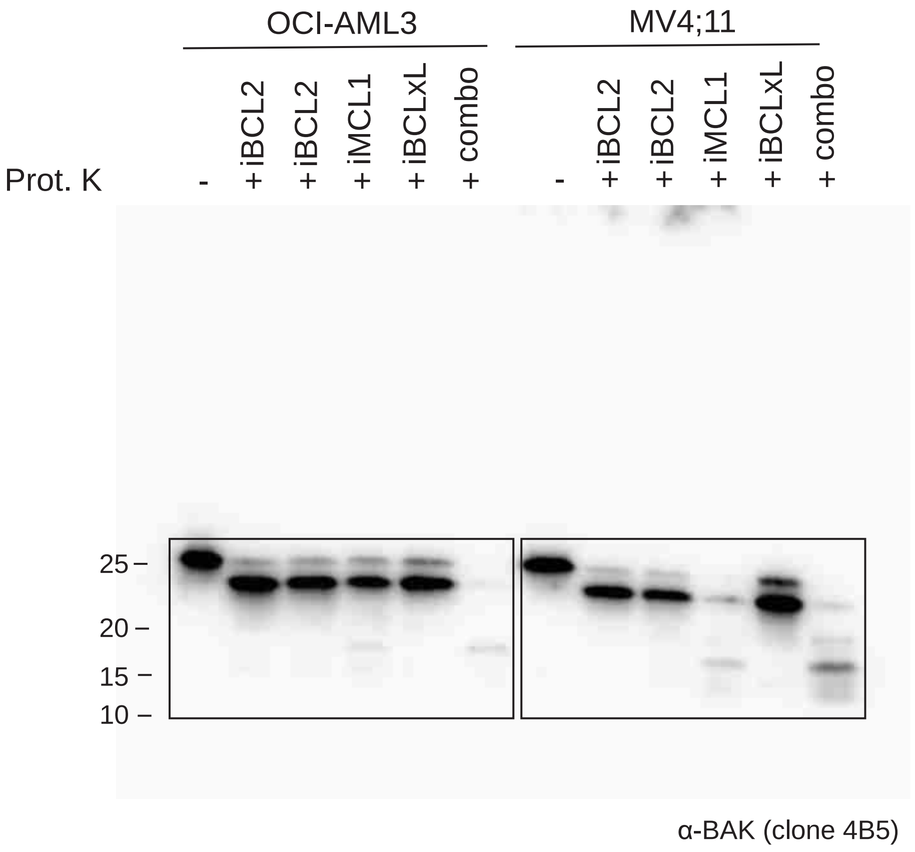

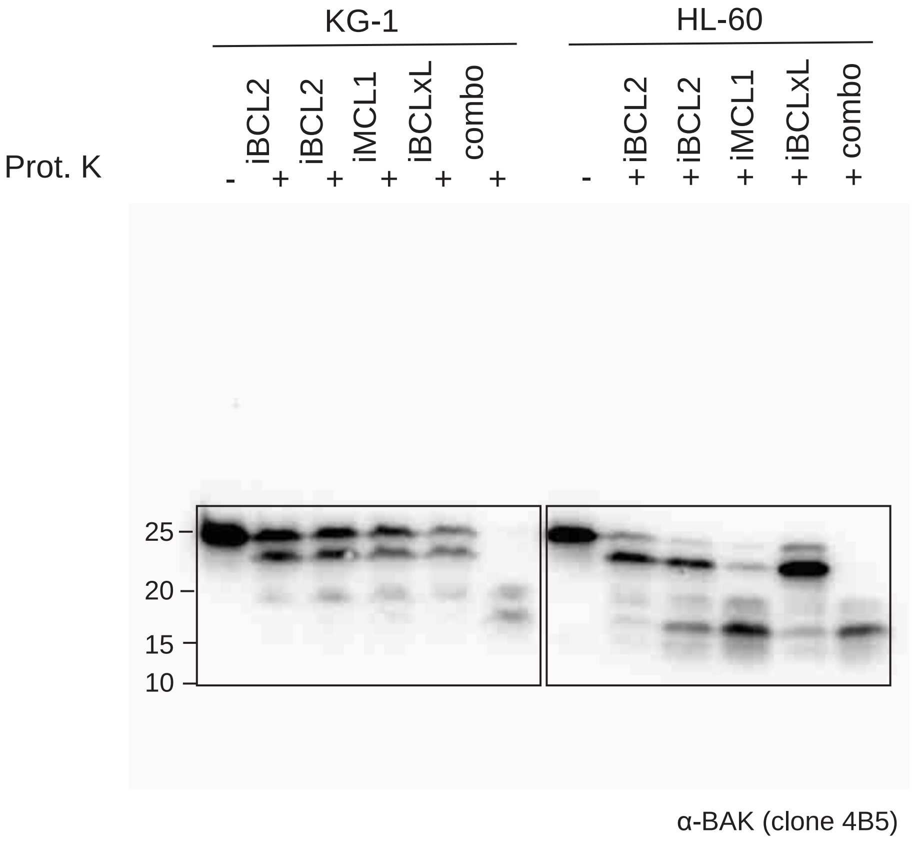

Figure S6 (BAX)

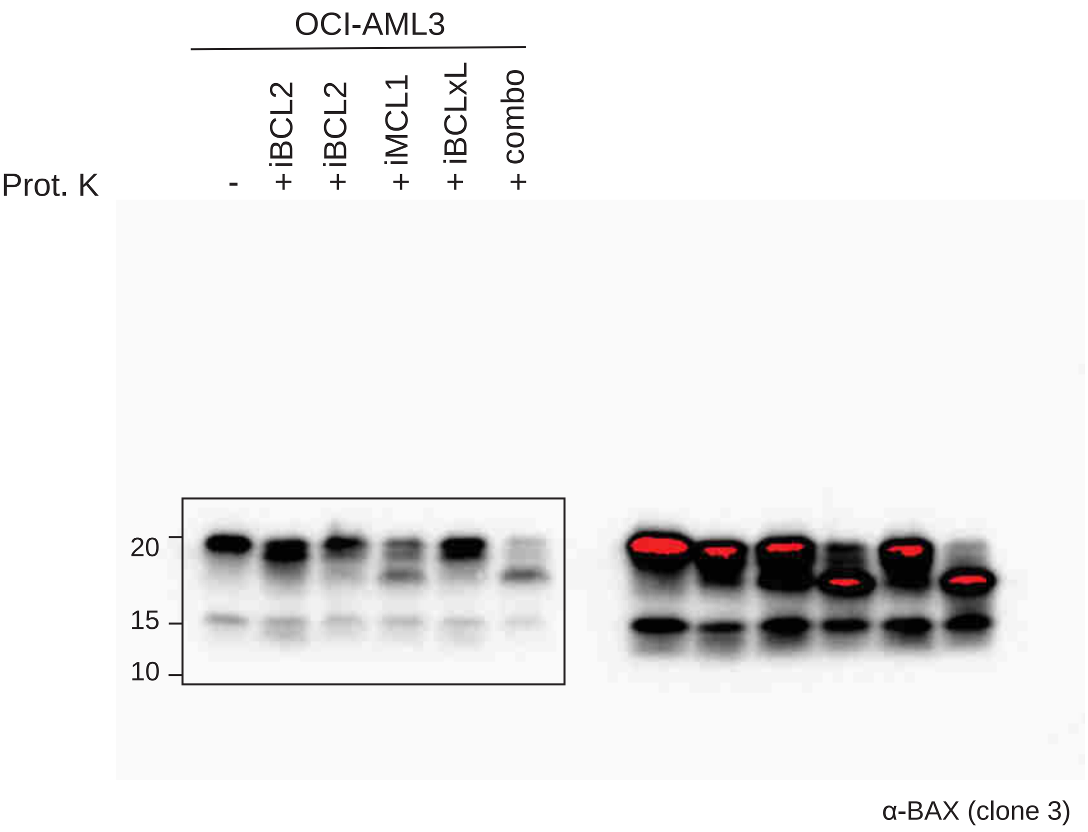

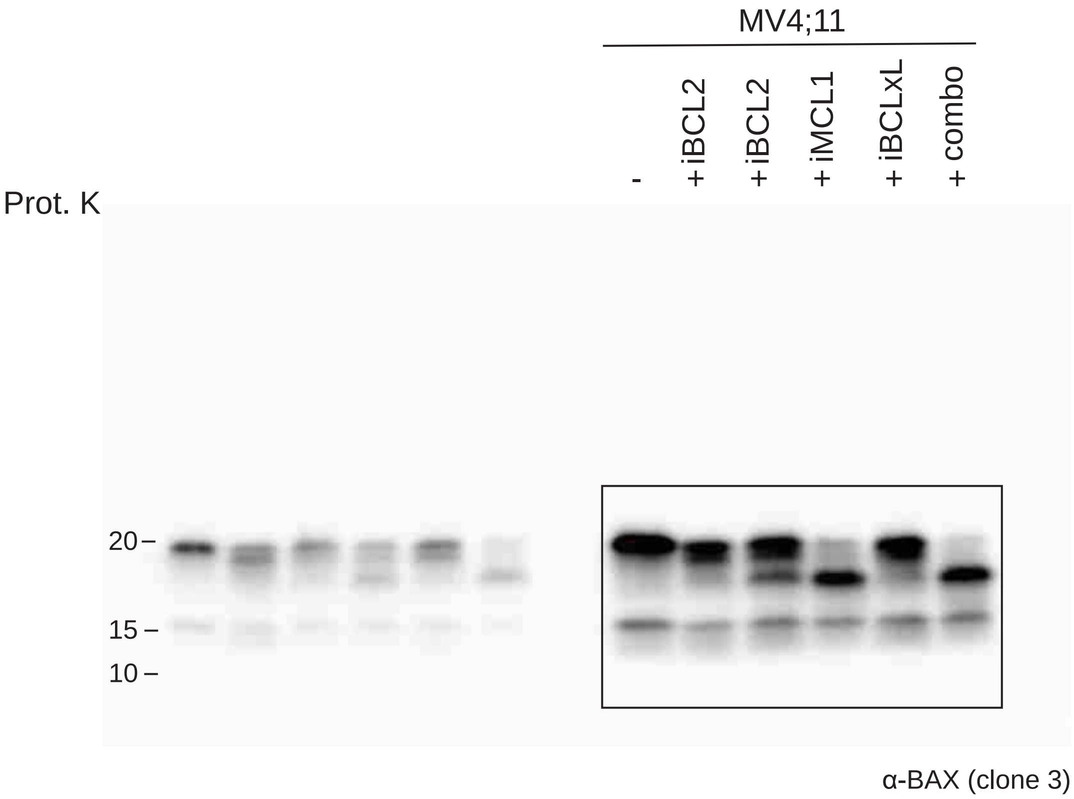

Figure S6 (BAX) Cont'd

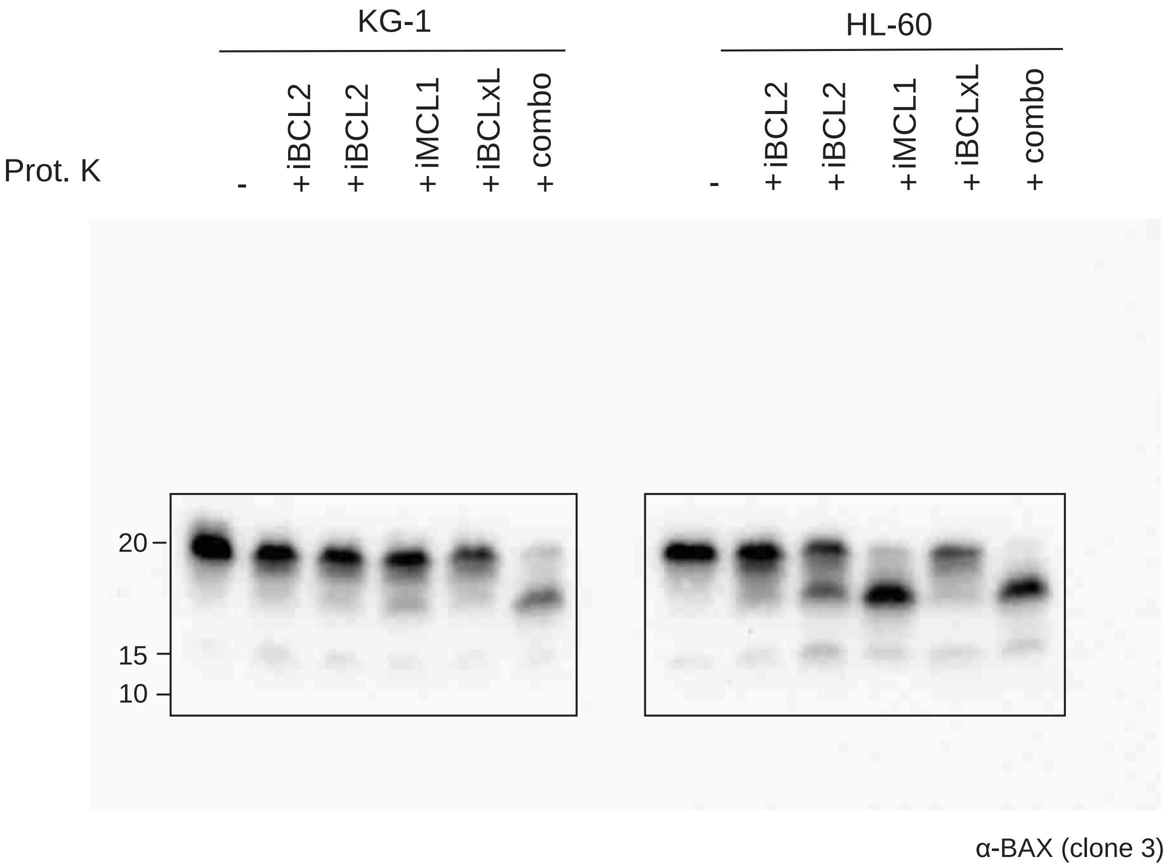

Supplement: Supplementary file 2 — Supplementary original blots [file 41418_2024_1289_MOESM2_ESM.pdf]
